# High-resolution characterization of ex vivo AAT polymers by solution-state NMR spectroscopy

**DOI:** 10.1126/sciadv.adu7064

**Published:** 2025-05-07

**Authors:** Sarah M. Lowen, Christopher A. Waudby, Alistair M. Jagger, Ibrahim Aldobiyan, Mattia Laffranchi, Annamaria Fra, John Christodoulou, James A. Irving, David A. Lomas

**Affiliations:** ^1^ UCL Respiratory, Rayne Institute, and the Institute of Structural and Molecular Biology, University College London, London WC1E 6JF, UK.; ^2^ UCL School of Pharmacy, University College London, London WC1N 1AX, UK.; ^3^ Department of Biochemistry, College of Science, King Saud University, PO Box 2455, Riyadh 11451, Saudi Arabia.; ^4^ Department of Molecular Medicine, Sapienza University of Rome, Laboratory Affiliated to Istituto Pasteur-Fondazione Cenci Bolognetti, Viale Regina Elena 291, 00161 Rome, Italy.; ^5^ Department of Molecular and Translational Medicine, University of Brescia, Viale Europa 11, 25123 Brescia, Italy.; ^6^ Department of Structural and Molecular Biology, and the Institute of Structural and Molecular Biology, University College London, London WC1E 6BN, UK.

## Abstract

Serpins, protease inhibitors whose regulated conformational instability renders them susceptible to mutations that cause misfolding, represent a system for the study of non-amyloid protein aggregation. The E342K “Z” variant of α-1-antitrypsin (AAT) undergoes oligomeric self-assembly into polymer chains that are associated with liver and lung pathologies in AAT deficiency. Structural characterization of polymers from human tissue has been limited by their heterogeneity and flexibility; here, we have studied their internal structure, which provides insights into the molecular linkage and the pathway by which they are formed. NMR spectra of heat-induced ^13^C-ILV-methyl–labeled polymers, and ^1^H-methyl spectra of liver-derived polymers, show equivalence to that of AAT in a post–protease-encounter conformation. This is corroborated by x-ray crystallography, which reveals a cryptic epitope recognized by the conformationally selective 2C1 antibody, common to both forms. These data definitively preclude most models of polymerization and are compatible with sequential intermolecular donation of the carboxyl terminus of one molecule into the next during polymer formation.

## Teaser

The intermolecular linkage of in vitro and ex vivo α-1-antitrypsin polymers is determined by solution-state NMR spectroscopy.

## INTRODUCTION

Serpinopathies are conditions that involve the aberrant behavior of members of the serpin superfamily of protease inhibitors. Serpins regulate diverse proteolytic pathways involved in processes such as hemostasis, tissue repair, immune function, and neural development, and their conformational pathologies have commensurate phenotypes including emphysema, thrombosis, angioedema, and dementia (*1*). Many of these conditions involve a distinct misfolding and non-amyloid self-assembly to form chains of oligomeric and polymeric molecules (“serpin polymers”) that have a flexible beads-on-a-string appearance by electron microscopy (EM). The association of these polymers with the development of disease and their unique properties make them important in the study of structural and cellular mechanisms of non-amyloid conformational pathologies (*2*–*6*).

The investigation of amyloidogenic processes has been advanced by the identification of conditions that promote amyloid formation in vitro, although some approaches result in morphologies that are not found in vivo (*7*–*9*). Thus, the study of aggregates isolated directly from pathological tissue has become a crucial nexus for the understanding of the molecular processes underlying these diseases. Because of the relative rigidity and the internal geometry of amyloid fibrils, recent advances in the structural characterization of native material have been possible by cryo-EM and solid-state nuclear magnetic resonance (NMR) (*10*, *11*). In contrast, serpin polymers present challenges to structural characterization due to the flexibility of the chains and apparent absence of regular, higher-order interactions (*2*–*4*, *12*). Furthermore, approaches such as the elongation of amyloid seeds from ex vivo material using isotopically labeled protein to undertake solid-state NMR spectroscopy (*13*) are not possible for serpins due to an absence of these amyloid-specific processes. The observation that polymers induced in vitro can be noncognate (*14*, *15*) makes the characterization of ex vivo serpin aggregates important for deriving an understanding of relevant molecular mechanisms that underlie their specific disease-related properties.

Our aim here has been to use solution-state NMR spectroscopy to obtain residue-specific information on the subunit structure and polymerization mechanism of a prototypical serpin, α-1-antitrypsin (AAT). AAT is a 52-kDa glycosylated serpin normally found at concentrations of 1 to 2 g/liter in human plasma, where its role is to protect tissue from damage by neutrophil-derived serine proteases, particularly neutrophil elastase (*1*, *16*). AAT uses the canonical serpin mechanism of protease inhibition. As with other serpins, AAT presents a bait sequence on an exposed structural element known as the reactive center loop (RCL). This RCL bait sequence is recognized and proteolytically attacked by the target, neutrophil elastase. Cleavage of a protein by a serine protease involves formation of a transient covalent ester linkage between the two molecules; for a normal substrate, the ester linkage then undergoes hydrolysis to release the cleaved product. However, with AAT, this step is interrupted by a structural rearrangement in which its RCL becomes inserted as an additional sixth strand within its central β-sheet A. This insertion is rapid enough that the hydrolysis of the ester bond does not occur, irreversibly trapping AAT and neutrophil elastase in a covalent complex (*1*, *17*).

Structurally, accommodation of the RCL as an additional strand into the central β-sheet of AAT involves hinge-like motions of rigid fragments, reorientation of some secondary structural elements, and repacking of the core (*18*). Polymerization is believed to be a byproduct of the ability of AAT and other serpins to undergo this conformational change, when destabilized by mutations or extrinsic experimental factors such as heat or denaturants (*1*, *16*, *17*, *19*, *20*). This process was first described for the E342K mutation of the Z variant (c.1096G>A, p.Glu366Lys), present in up to 1 in 40 northern Europeans (*1*, *16*, *17*), which in homozygosity causes severe AAT deficiency (AATD) and accounts for over 95% of cases of this condition (*1*, *16*, *21*). It has long been recognized that inclusions form in the liver tissue of individuals homozygous for the Z allele (*22*, *23*); these were eventually determined to be composed almost exclusively of AAT polymers (*2*). The retention of AAT polymers in the endoplasmic reticulum of hepatocytes, coupled with a high degradative turnover of a large proportion of misfolded protein by cellular quality control machinery, directly causes the low levels of circulating functional AAT in plasma. The consequent unregulated neutrophil elastase activity predisposes affected individuals to early-onset emphysema (*1*, *16*, *24*).

**Fig. 1.**
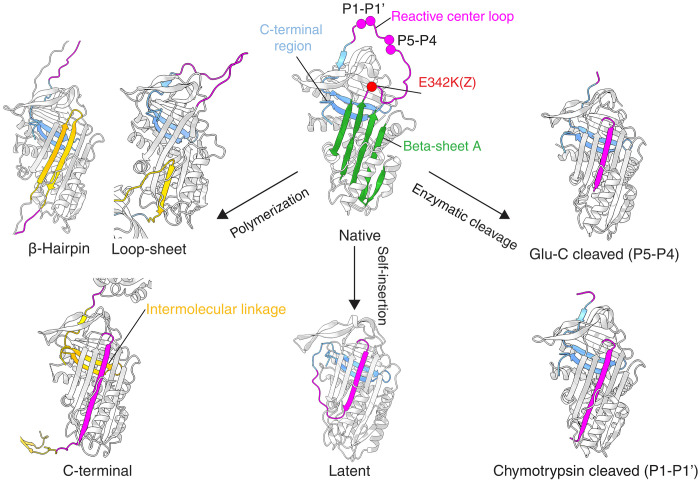
Native and inserted AAT structures and polymerization models. Native AAT crystal structure (PDB: 8PI2) with the C-terminus shown in blue, β-sheet A in green, and RCL in purple. The Z AAT E342K mutation site is highlighted as a red sphere. The enzymatic cleavage sites relevant to the present work at the canonical peptide bond cleaved by chymotrypsin [PDB: 1EZX (*51*)] and neutrophil elastase and cleaved by Glu-C at the P5-P4 bond (PDB: 9GGP; reported here) are shown highlighted as purple spheres on the native RCL. Partial self-insertion of the RCL leads to the latent AAT structure [PDB: 1IZ2 (*48*)]. The loop-sheet (*2*), β-hairpin (*26*), and C-terminal (*37*) polymerization models are shown, with the intermolecular linkages highlighted in yellow. The figure was prepared using ChimeraX (*80*).

The structural changes that occur during polymer formation in human tissue remains a crucial and unsolved question. Several hypotheses have been advanced regarding the structure of the pathological AAT polymer (Fig. 1), based on biophysical and structural data in various studies that did not directly address the relevance to polymers that form in human tissue. We have shown that molecular contours of the repeating polymer subunit, reconstructed from negative-stain EM (NS-EM) images of liver tissue–derived polymers, are most compatible with a linkage mediated by intermolecular donation of a C-terminal region (*4*). However, direct evidence of the internal structural details of the liver-derived polymer subunit remains lacking, including whether its central β-sheet A is in a six-stranded conformation. This information is key to the rational development of therapeutics to interfere with polymerization, diagnostics to follow its progression, and the question of the feasibility of reversing this process.

We previously used solution-state NMR to study the monomeric conformation of AAT purified from plasma of patient donors using two-dimensional (2D) ^1^H,^13^C experiments at natural isotopic abundance and identified a monomeric and pathologically important intermediate associated with early stages of the polymerization pathway (*25*). Here, we report the structural characterization of the polymers that result, including those isolated from liver tissue of individuals who have undergone transplantation for cirrhosis related to AATD. In tandem, we have determined, by x-ray crystallography, a cryptic epitope that is common to liver-derived polymers and heat-induced polymers. In combination, these data show that the subunit of artificially induced and pathologically occurring polymers is in a six-stranded β-sheet A configuration, with a fully self-incorporated RCL. These data preclude several models of polymerization (*2*, *19*, *26*–*28*) and, in the context of the periodicity and flexibility of Z AAT polymers, are only consistent with an asymmetric linkage mediated through sequential intermolecular donation of the C-terminus from one subunit to the next. This represents a rare example of a pathogenic aggregation mechanism that essentially recapitulates a folded state encountered during normal function of the protein. Thus, the energetic barrier that the Z variant overcomes during production in hepatocytes is not one between “native” and “non-native” but between functional start and end states and explains the inability of the cellular machinery to recognize the resulting polymers as misfolded.

## RESULTS

### High-resolution 2D NMR spectroscopy of purified recombinant AAT polymers

AAT polymer chains generally range in size between dimers (0.1 MDa) and 20-mers (1.0 MDa) (*4*, *29*). Methyl groups are well-suited to the characterization of large molecules by NMR spectroscopy, leveraging the methyl TROSY effect to reduce relaxation (associated with increasing molecular weight) and increase sensitivity and resolution (*30*, *31*), allowing characterization of systems up to the megadalton scale (*30*, *32*–*35*). We have previously used this approach to study the structure and dynamics of monomeric AAT variants (*25*). The most common methyl-containing side chains are isoleucine, leucine, and valine (ILV), which comprise 22% of residues within AAT; given the sensitivity of methyl chemical shifts to the structure and dynamics of their surrounding environment [out to ~5.5 Å (*36*)], this provides coverage of changes in structure and dynamics across essentially the entire molecule.

The characterization was undertaken in a hierarchical manner; initially, we used recombinant ^13^C isotopically labeled protein in defined conformational states—in the native conformation, cleaved by a protease, or induced to form polymers—before extending our analyses to clinical samples derived from human plasma and liver tissue.

**Fig. 2.**
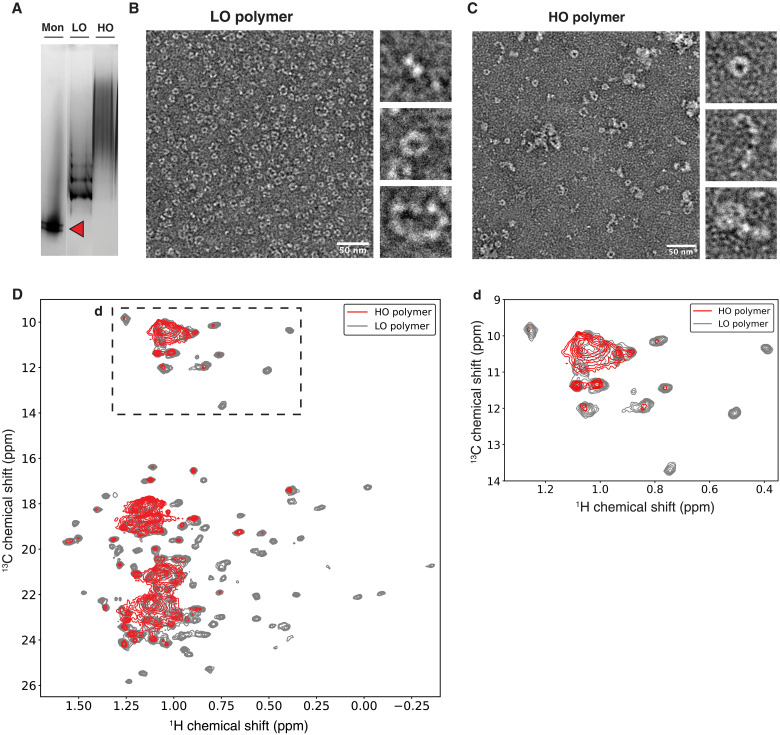
Purification of heat-polymerized AAT. (**A**) Three percent to 12% (w/v) acrylamide nondenaturing gel showing heated AAT as laddered material and HO diffuse polymers; 4 μg of protein was loaded in each lane, with plasma Z AAT monomer used as a reference (red arrow). (**B**) Representative NS-EM image of LO polymers; analysis of two images revealed molecules to be almost entirely circular, with these self-terminating species corresponding with trimer (15%), tetramer (50%), pentamer (23%), and hexamer (11%). Scale bar, 50 nm. (**C**) Representative NS-EM image of HO polymers; circular polymers (52%) ranged in size from dimer to hexamer with a preference for tetramers; linear polymers (36%) ranged in size from tetramer to nonamer with a predominance of hexamers, and aggregates (12%) were also evident. Scale bar, 50 nm. (**D**) ^1^H,^13^C HMQC spectra overlay of LO polymer (gray), 25 μM (328 K, 950 MHz) and HO polymer (red), 41 μM (328 K, 950 MHz). Contour levels were normalized for effective concentration of polymer subunits. Upper right panel (d) is a close-up of the Ile resonances.

To prepare a sample of recombinant AAT polymers incorporating ILV isotopic labeling for comparison with clinical material, we incubated recombinant protein at 328 K for 48 hours (fig. S1A) and used ion exchange chromatography (IEC) to remove any residual monomer. In the process, two polymer populations were resolved: lower-order (LO) polymers that formed laddered bands on a nondenaturing polyacrylamide gel electrophoresis (PAGE) gel and higher-order (HO) polymers that could not be resolved individually (Fig. 2A). NS-EM (Fig. 2, B and C) showed the LO fraction to be dominated by circular polymers, while the HO fraction was a heterogeneous mixture with around half circular polymers, a third linear, and the remainder appearing as condensed aggregates. The circular forms are reminiscent of a subcomponent of a mixture chromatographically enriched from a denaturant-treated disulfide mutant of AAT and also produced by cytoplasmic expression in *Pichia pastoris* (*37*, *38*). We have previously shown that heat treatment of wild-type AAT (M AAT) from plasma generates a greater proportion of circular polymers than are found in liver tissue (*4*). It is not possible to confidently ascribe these morphological differences to alternative intermolecular contacts between subunits; they could, for example, arise from context-specific differences in the rate of polymer elongation by polymer-polymer concatenation relative to monomer addition.

2D ^1^H,^13^C heteronuclear multiple-quantum coherence (HMQC) correlation spectra were acquired for both LO and HO polymer fractions separately. Acquisition at elevated temperatures (328 K) is often used in NMR to improve resonance sensitivity and resolution for large species by increasing the motional narrowing of NMR resonances. No changes in spectra were observed during the data acquisitions (fig. S2), indicating that samples remained stable. The spectrum of the LO polymers showed generally well-defined resonances and overlaid extensively with those resolvable in the HO fraction (Fig. 2D), suggesting that the individual AAT subunits found in circular and linear polymers were structurally similar overall.

Resonances within the HO polymer spectrum were more poorly resolved, with large variations in intensity and linewidth, resulting in regions of extensive peak overlap. The strongest resonances were observed in regions close to random coil chemical shifts, which is likely correlated with increased local mobility counteracting the effect of high molecular weight. It is also possible that these resonances may reflect some inhomogeneous broadening associated with structural heterogeneity, such as through inter-polymer or inter-subunit interactions.

### Comparison of cleaved and polymeric AAT

**Fig. 3.**
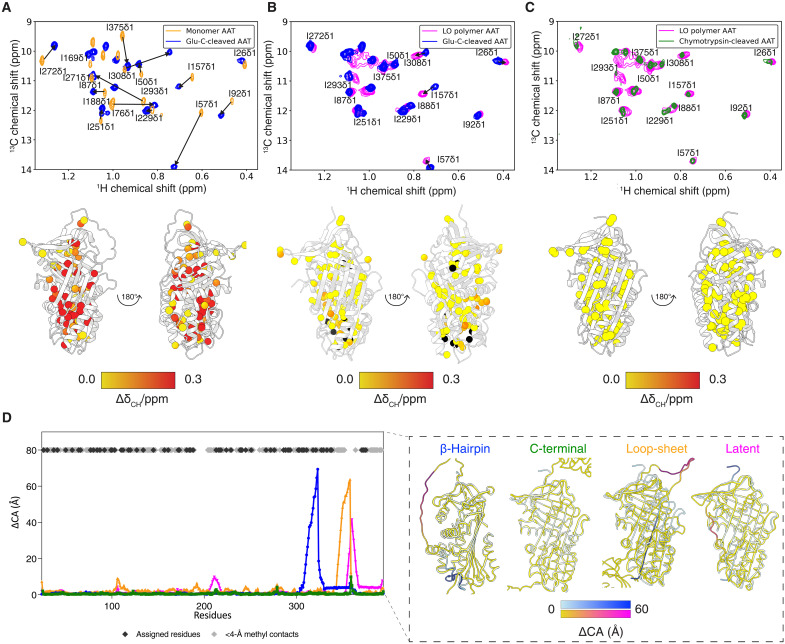
Comparison of monomer, cleaved, and polymeric AAT. (**A**) Overlay of ^1^H,^13^C HMQC spectra of monomer (orange) and Glu-C–cleaved AAT (blue) (ILV labeling, 328 K, 950 MHz). (**B**) Spectral overlay of ^1^H,^13^C HMQC spectrum of Glu-C–cleaved AAT (blue) and LO heat polymer (magenta) (328 K, 950 MHz). (**C**) Spectral overlay of ^1^H,^13^C HMQC spectrum of LO heat polymer (magenta) and chymotrypsin-cleaved AAT (green) (328 K, 950 MHz). Combined methyl shift changes are shown below each respective spectrum, $\Delta \delta_{\text{CH}}=\sqrt{\Delta {\delta^{2}}_{H}+{\left(\frac{\Delta \delta_{C}}{4}\right)}^{2}}$, projected onto the native (PDB: 8PI2) and cleaved AAT (PDB: 9GGP; reported here) structures, respectively, with observed methyl groups shown as colored spheres representing the CSPs with a gradient from Δδ 0.0 ppm (yellow) to Δδ 0.3 ppm (red), depicted using ChimeraX (*80*). Black spheres are resonances that could not be reliably transferred due to spectral overlap or drastic chemical shifts. (**D**) Distances (Å) between equivalent Cα residues in comparisons of the fully inserted RCL form of AAT [PDB: 1EZX (*51*)] with different polymer models (*4*) and latent AAT [PDB: 1IZ2 (*48*)]. Black and gray diamonds represent chymotrypsin-cleaved AAT residues that have been assigned or are within 4-Å contacts, respectively.

One feature common to most models that have been proposed for the AAT polymer is a subunit in which β-sheet A is in a six-stranded configuration (*2*, *4*, *26*, *37*). This is similar to the structure of the protease-cleaved form of the protein (Fig. 1). To explore this possible correspondence, we prepared uniformly ^13^C,^15^N-labeled samples of recombinant AAT cleaved within the RCL by the tool protease Glu-C (fig. S3). Cleaved AAT is extremely thermostable; with the increased sensitivity and resolution available at higher temperatures, backbone amide and side-chain methyl resonances were successfully assigned at 321 K with no need for deuterated labeling schemes (figs. S4 and S5) [Biological Magnetic Resonance Data Bank (BMRB) ID: 52599]. This revealed widespread and large chemical shift perturbations (CSPs) relative to the uncleaved native state (Fig. 3A). By contrast, the Glu-C–cleaved AAT spectrum corresponded closely with that of the polymer (Fig. 3B), providing strong evidence for a shared structure.

Methyl resonance assignments were then transferred to the polymer using our previous strategy, in which resonances showing smallest perturbations were attributed first, followed by those with larger CSPs while avoiding outliers in disconnected regions of the structure (*25*). The Glu-C protease is a useful tool by virtue of its lack of stable complex formation with AAT, which is the result of cleavage at the atypical P_5_-P_4_ site in the RCL (*39*). This produces a self-inserted RCL that is four residues shorter than seen with AAT cleaved by chymotrypsin and neutrophil elastase in the canonical P_1_-P_1_′ position (illustrated in Fig. 1). Therefore, an alternative RCL-cleaved form of AAT was also prepared using chymotrypsin. Chromatographic removal of the inhibitory complex yielded enough cleaved material to obtain a 2D ^1^H,^13^C HMQC correlation spectrum (Fig. 3C). Demonstrating the sensitivity of this technique to small perturbations in structure, the four amino acid difference in cleavage site was reflected by small CSPs [up to 0.3 parts per million (ppm)] in two clusters sandwiching the base of β-sheet A (fig. S7, A and C). As a consequence, the chymotrypsin-cleaved material provided resonances that overlaid near perfectly with that of the polymer, including in this region (Fig. 3C and fig. S7, B and D).

These experiments revealed equivalence in structure, contacts, and dynamics in the vicinity of the methyl probes between cleaved AAT with a fully self-incorporated RCL and heat-induced AAT polymers. The spectra encompass 95 methyl moieties of ILV side chains, residue types that predominantly are involved in intramolecular packing within the protein core. Crucially, this equivalence was unequivocal for all secondary structural elements. Thus, it could be concluded that the heat-induced AAT polymer subunit has a fully self-incorporated RCL and a six-stranded β-sheet A configuration.

### X-ray crystal structure of 2C1 Fab in complex with cleaved AAT

Both heat-induced polymers and the polymers that form in liver tissue have a common 3D shape and flexibility (*4*). Additionally, they expose a cryptic epitope absent in native and denaturant-treated material that is recognized by the 2C1 monoclonal antibody (mAb2C1) (*14*, *15*, *40*). This antibody has provided an important nexus between in vitro induced and naturally-arising polymers (*6*, *41*, *42*), and its specificity established by a range of studies and clinical samples (*5*, *40*, *43*). We therefore sought to identify the structural details of its epitope.

**Fig. 4.**
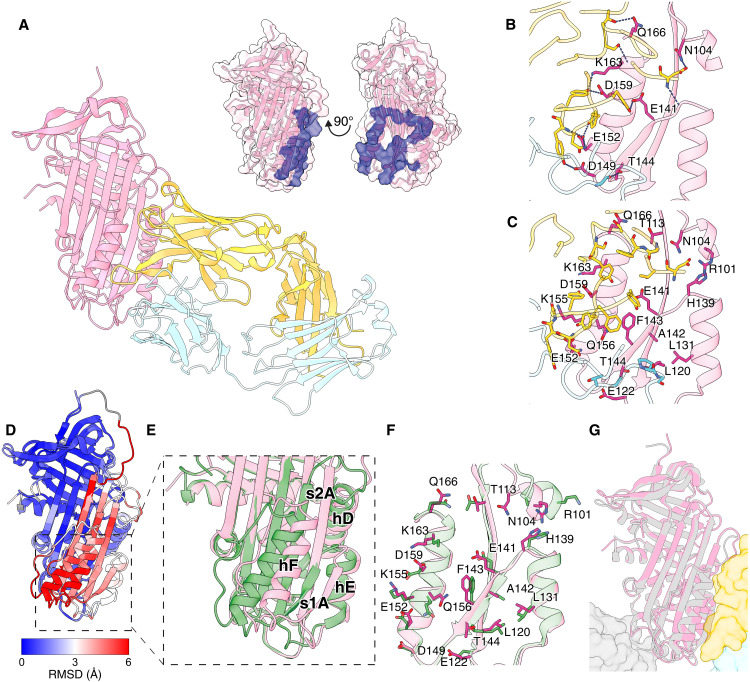
The Fab fragment of anti-polymer monoclonal antibody, 2C1, recognizes the cleaved form of AAT. (**A**) Crystal structure of Fab2C1, with the heavy and light chain shown in yellow and blue, respectively, in complex with Glu-C–cleaved AAT shown in pink (PDB: 9GGP; reported here). At the top right, the AAT epitope surface is highlighted in blue. (**B**) H-bonds and (**C**) van der Waals contacts at the Fab2C1 and AAT interface are shown as stick models. Labels are included for all AAT residues that interact with Fab2C1. Hydrogen bonds are shown as dashed lines. (**D**) Native AAT (PDB: 8PI2) and cleaved AAT from the 2C1 complex are superimposed from residues 194 to 393 and colored by root mean square deviation (RMSD) (blue, white, red). (**E**) Close-up view of the epitope region on cleaved AAT (pink) in comparison with the native state (green), highlighting secondary structure shifts resulting from RCL insertion. (**F**) Native and cleaved AAT aligned at the Fab2C1 interaction site (residues 100 to 167) with all residues involved in Fab2C1 binding shown as stick models. (**G**) Protease (gray surface)–bound AAT with fully inserted RCL (gray cartoon) [PDB: 1EZX (*51*)] in comparison with Glu-C–cleaved AAT (pink cartoon) in complex with Fab2C1 (heavy shown as yellow surface) (PDB: 9GGP; reported here) (RMSD, 0.78 Å).

The Fab fragment of mAb2C1 was incubated with Glu-C–cleaved AAT, the complex was isolated by size exclusion chromatography, and crystallization trials yielded a crystal that diffracted to 1.84 Å.The solved structure (Fig. 4A and Table S1) revealed the epitope to be defined by helix E, helix F, the upper portion of helix D, and β-strands 1A and 2A. This is a site intimately associated with the transition between the five- and six-stranded β-sheet A configurations of AAT; upon RCL insertion, this region is displaced as a fragment with respect to the rest of the molecule, with compensatory rotation of helix F and loss of surface-accessible cavities (Fig. 4, D and E) (*44*–*47*). The 993-Å^2^ intermolecular interface between AAT and Fab2C1 involves 12 hydrogen bonds and 101 van der Waals contacts (Fig. 4, B and C). Superposition of the native AAT molecule with the cleaved conformation within residues 97 to 167, delineated by the epitope, showed steric clashes with the N-termini of helices D and F and reduced contacts with β-strands 1A and 2A as the likely basis for selective antibody recognition of the inserted conformation. When compared with the crystal structure of the fully loop-inserted, protease-bound conformation, binding by Fab2C1 was confirmed to not have induced further changes in AAT (Fig. 4G). The recognition of heat-induced and liver-derived polymers by this antibody therefore indicates that, for both samples, helices D, E, and F and β-strands 1A and 2A are intact and properly folded, and in a configuration consistent with a six-stranded β-sheet A.

### Heat-induced polymers are mediated by a C-terminal intermolecular linkage

The NMR data provide the first confirmation that subunits of heat-polymerized AAT are structurally equivalent to the fully RCL-inserted conformation. The spectral overlap with RCL-cleaved AAT further confirms that the heat polymers are essentially homogeneous for a single structural form whose secondary structural elements are intact. This is consistent with the structure of the cryptic epitope recognized by mAb2C1.

Unlike serpins such as plasminogen activator inhibitor-1, it is not possible for both the shorter RCL of AAT to be fully self-inserted and for the molecule to have a self-incorporated C-terminus, as evident in the incomplete insertion seen with the monomeric latent form (*48*) (Figs. 1 and 3D). Thus, for the subunits to be fully formed, the RCL and C-terminus (comprising β-strands 1C, 4B, and 5B) must be incorporated into adjacent molecules in trans, with the intervening residues acting as an intermolecular linker (Figs. 1 and 3D).

The presence of the fully-inserted RCL is inconsistent with the loop-sheet mechanism, whose intermolecular contact is mediated by an RCL only partially incorporated into the β-sheet A of an adjacent molecule (Fig. 3D, illustrated in Fig. 1). This represents a special case of a “double-intermolecular-linkage” polymer configuration; all other variations on such an arrangement, such as the β-hairpin model in which helix I and succeeding residues are unwound, require loss of one or more secondary structures to provide sufficient flexibility to accommodate the second linkage. Given the fidelity with which the heat-induced polymer corresponds with an intact cleaved conformation, and that cleaved AAT is a structural mimetic of a subunit of polymers formed through the C-terminal mechanism, it can be concluded that spectra of heat-perturbed AAT polymers are only consistent with a single C-terminal intermolecular linkage (Fig. 1).

### The subunit of Z AAT polymers formed in human tissue corresponds with the full RCL–incorporated conformation

**Fig. 5.**
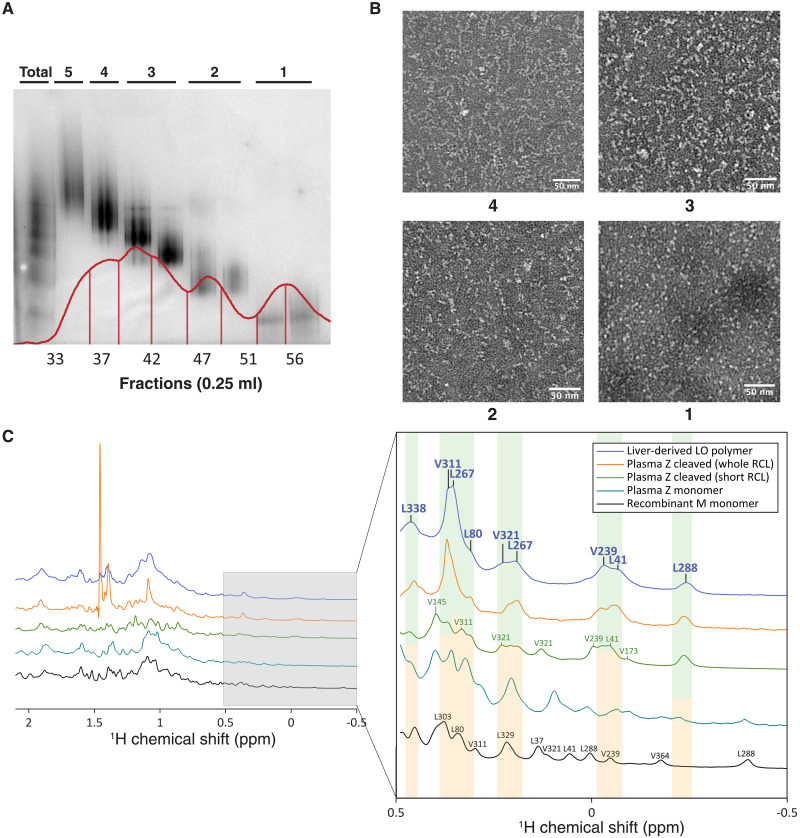
Purification of Z AAT polymers from explanted human liver. (**A**) Nondenaturing PAGE analysis of liver-derived AAT polymers after final separation by size exclusion chromatography. The corresponding elution chromatogram is overlaid in red. Each lane has ~4 μg of protein. (**B**) NS-EM imaging of the fractions of liver polymers. Scale bar, 50 nm. (**C**) ^1^H 1D NMR spectra (328 K, 950 MHz, and 1 GHz) and close-up of the high-field methyl region of liver-derived LLO polymers (purple) and reference proteins: plasma Z AAT cleaved at the RCL with chymotrypsin (full RCL; orange), plasma Z AAT cleaved at the RCL with Glu-C (short RCL; green), monomeric plasma Z AAT (teal), and recombinant M monomer (black). Resonances from the liver-derived sample, assigned by spectral alignment, are shown in purple. Green and yellow panels indicate reference spectra high-field methyl resonances that do and do not correspond with the liver spectra, respectively.

We sought to extend our investigation to the pathological polymers of Z AAT that form in the human liver. Tissue was obtained from an individual homozygous for the Z allele who had undergone transplantation for AATD-associated liver failure. Polymers were extracted and subjected to chromatographic separation (immobilized metal affinity, ion exchange, and size exclusion chromatography) to obtain a series of fractions of pure AAT with a progression in chain size (Fig. 5, A and B, and fig. S8).

As mentioned above, polymers are highly thermostable, and preliminary experiments with incubation at 328 K showed a lack of aggregation or further polymerization (fig. S9). To mitigate size-dependent loss of spectral resolution, the smallest species encapsulating the polymer linkage, the liver-derived low order (LLO) fraction enriched for dimers and trimers, was used. This permitted acquisition of ^1^H 1D spectra at this temperature and high field (950 MHz) using band-selective excitation of methyl groups following the principles of SOFAST-HMQC (*49*), which in combination provided the greatest resolution and sensitivity we were able to achieve (figs. S10 and S11). No time-dependent changes in methyl chemical shifts were observed over the maximal 14-hour collection time, and a post-acquisition nondenaturing gel confirmed that no degradation, polymerization, or aggregation had taken place.

The optimizations in sample quality and collection parameters resulted in well-resolved, distinct high-field methyl resonances of the ^1^H 1D spectra of the LLO polymers. Reference ^1^H 1D NMR spectra were also collected for recombinant monomeric M AAT and monomeric Z AAT purified from the plasma of individuals, both in the native state and cleaved with Glu-C (producing a truncated inserted RCL) and chymotrypsin (producing a fully inserted RCL) (Fig. 5C). Notably, in common with their characterization by 2D ^13^C,^1^H spectra, these two alternative cleaved forms exhibited distinctive differences in methyl resonances despite a difference of four inserted RCL residues.

An examination of the liver-derived LLO spectrum showed closer correspondence with the spectrum of chymotrypsin-cleaved Z AAT than with Glu-C–cleaved or native states. These spectra are effectively 1D projections of 2D spectra, and due to the well-dispersed nature of the resonances at low chemical shift values, it was possible to transfer the original ^13^C,^1^H resonance assignments of recombinant cleaved wild-type AAT to the ^1^H spectra of cleaved Z AAT, and thereby to the liver-derived dimer spectrum. This was only done where peaks were distinct and could be reliably identified (fig. S12).

**Fig. 6.**
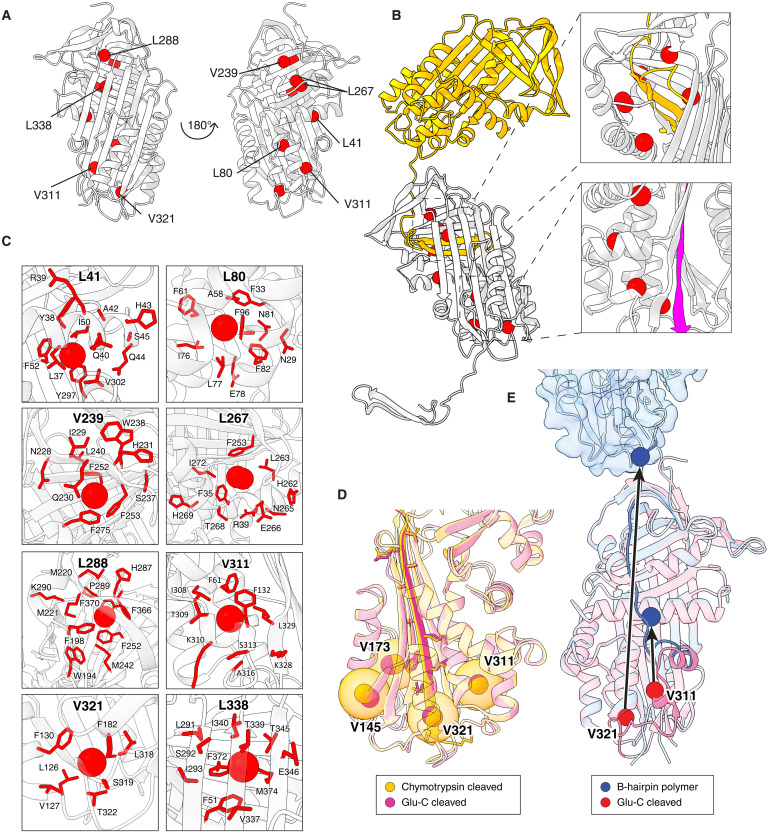
NMR spectroscopy of Z AAT polymers from explanted liver. (**A**) Assigned liver polymer methyl peaks mapped onto the structure of cleaved AAT [PDB: 1EZX (*51*)] shown as red spheres. (**B**) Left: Proposed C-sheet linkage of AAT polymers (*4*) with the donor molecule shown in yellow. Right: Close-up views of the inserted C-terminus (yellow) and RCL (purple) with assigned residues from the methyl 1D assignment of Z AAT polymers from explanted liver shown as red spheres. (**C**) Sidechains of residues within the local chemical environment (<4 Å) of those that are assigned in the 1D spectra. (**D**) Residues with resonances displaced in the Glu-C 1D spectrum compared to liver polymer and chymotrypsin-cleaved AAT shown as spheres for chymotrypsin-cleaved (orange) [PDB: 1EZX (*51*)] and Glu-C–cleaved AAT (pink) [PDB: 9GGP; reported here] with a 5-Å radius shown as a transparent orange sphere. (**E**) Cleaved AAT [PDB: 1EZX (*51*)] (pink) superposed with a representation of polymer AAT formed by the β-hairpin mechanism (blue). Secondary structures that are required to unfold to accommodate β-hairpin intermolecular linkage are shown in darker blue and pink. Residues V321 and V311 are highlighted as red and blue spheres on the cleaved and β-hairpin polymer structures, respectively, with arrows showing extent of structural change. Representations by ChimeraX (*80*).

Spectral crowding at higher ^1^H chemical shifts limited the number of methyl groups that could be assigned. Nevertheless, the methyl resonances with low chemical shift values that could be resolved were well dispersed throughout the molecule (Fig. 6A). The close correspondence between the spectrum of the liver-derived sample and that of chymotrypsin-cleaved Z AAT, and the comparative distinction with Glu-C–cleaved and native spectra, indicates that the residues are in a very similar local chemical environment and therefore molecular structure. This chemical environment extends beyond each respective residue to include contacts reported by adjacent residues within a 4-Å proximity, and 5.5 Å for aromatic residues (*36*) (shown as red sticks in Fig. 6C).

The 1D spectra of Glu-C–cleaved AAT notably differed to chymotrypsin-cleaved and liver AAT by CSPs in residues V321, V173, V145, and V311 (highlighted in Fig. 5C), despite the position of these residues in the Glu-C– and chymotrypsin-cleaved AAT crystal structures appearing very similar (Fig. 6D). This reflects the sensitivity of NMR to differences in conformational sampling in solution that are not observable in the context of the crystallographic lattice. The close proximity of these residues to the base of β-sheet A makes the difference in 1D spectra likely to be a consequence of the difference in RCL length.

According to the β-strand 5A/RCL hairpin model of polymerization, inter-subunit interactions require unraveling of helix I and the adjacent region to allow for a flexible intermolecular linkage with sufficient length (*26*). Residues V311 and V321, identified from the liver polymer 1D spectra by correspondence with chymotrypsin-cleaved AAT, are located in helix I and the post-helix I loop, respectively. If the liver polymers were mediated by a β-hairpin exchange, these residues would be in markedly different chemical environments that would result in substantial CSPs in the 1D spectra; however, the data do not show this (Fig. 6D).

**Fig. 7.**
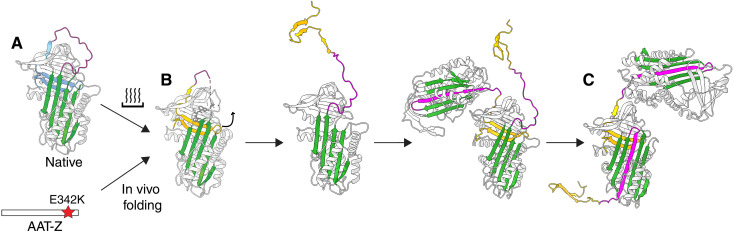
Schematic of the proposed C-terminal mechanism showing convergence of heat-induced polymerization and Z AAT folding in vivo to the early-stage intermediate [PDB:7AEL (*42*)] through intermolecular linked polymer chains. States along the polymerization pathway that have been directly observed by NMR spectroscopy by previous studies: (**A**) native AAT, (**B**) a polymerization intermediate state, and by this study: (**C**) full RCL incorporation. The prevailing evidence is that polymerization occurs from a near-native intermediate (B) (*42*) and that the C-terminus (yellow) must be incorporated before RCL self-insertion is able to occur (*64*) through the entirety of the central β sheet (C). Representations by ChimeraX (*80*).

Thus, as the assigned probes included some situated directly behind the base of β-sheet A, where the RCL is inserted, and residues surrounding the inserted C-terminus (shown as purple and yellow, respectively, in Fig. 6B), this allowed us to conclude that liver-derived polymer subunits are most structurally consistent with full RCL–incorporated Z AAT (Fig. 6B). As observed for the heat-induced polymers, due to the structural constraints imposed by RCL loop length and intact-secondary structures, this is only compatible with incorporation of the RCL and C-terminus occurring in trans, and thereby a C-terminal domain swap-mediated polymer configuration (Fig. 7). The assigned spectra are directly incompatible with the β-hairpin and loop-sheet models or the latent conformation. In addition, in the context of the above-reported mAb2C1 epitope, these data are also incompatible with a latent- or native-like protomer as proposed by the C-sheet and edge-strand models.

## DISCUSSION

In the time since our characterization of patient tissue–derived inclusion bodies as comprising linear chains of AAT molecules (*2*), few studies have directly investigated the structural characteristics of naturally occurring polymers. We have shown that soluble polymers are present in lung lavage fluid and are resistant to unfolding in 8 M urea (*50*) in common with heat-induced polymers (*39*), and that liver-derived polymers present a cryptic epitope (*15*, *40*), and we have reconstructed the 3D contour of constituent dimeric subunits within liver-derived polymers using NS-EM (*4*). We have also previously used 2D NMR spectroscopy at natural isotopic abundance to study AAT purified from human plasma (*25*). Further analysis was able to associate these changes with rapid exchange between the native state and an intermediate that could be stabilized by binding of a small-molecule ligand (*25*). While the sensitivity of the technique is affected by samples at natural isotopic abundance, it nevertheless has demonstrated potential to obtain data on structure and dynamics for otherwise intractable samples at a residue-specific level. Here, we have circumvented the low natural isotopic abundance of ^13^C and ^15^N by using ^1^H 1D measurements of ex vivo AAT polymers to obtain structural fingerprints using solution-state NMR spectroscopy. We focused our analyses on well-resolved resonances within the methyl region as probes for the liver polymer structure relative to a series of reference samples, which were well dispersed through the molecule.

Central to the polymerization of AAT is its ability to undergo conformational change, which is required for its inhibitory activity (*51*–*54*). This change involves incorporation of the protease-cleaved RCL as an additional central strand in β-sheet A, with accompanying alterations in the packing at the core of the molecule and adjustments in the position of secondary structural elements, resulting in a pronounced stabilization of the molecule. In polymers induced artificially from native material, such a stabilization is also seen upon transition to the polymeric state and with incubation of peptide mimetics of the RCL of various lengths (*2*, *39*, *55*, *56*). Consistently, some—although not all—proposed models of polymerization hypothesize a subunit with an expanded six-stranded β-sheet A and different extents of RCL incorporation (Fig. 1) (*4*, *57*, *58*). Previous biophysical, biochemical, and structural studies have largely been based on perturbation of native AAT in vitro, and one confounder has been that perturbation by heat or denaturant produces different polymer types, with elevated temperature recapitulating a state, at the level of molecular shape and epitope display, more consistent with ex vivo material (*4*, *14*). Our characterization of a cryptic epitope recognized by the mAb2C1 antibody and exposed in both heat-induced, liver-derived, and plasma-derived polymers (Fig. 4) has demonstrated that it occurs in a region of the molecule known to be associated with the transition of β-sheet A to a six-stranded conformation during RCL insertion (*18*, *59*–*61*).

Exploiting the sensitivity of solution-state NMR spectroscopy to small perturbations of structure and dynamics, we have shown that subunits of one population of heat-induced polymers is structurally identical to the cleaved monomer. In particular, the lack of CSPs in the inserted RCL and surrounding β strands between the cleaved and polymeric species (Fig. 3C and fig. S7) indicates that both share an “open” β-sheet A and full insertion of the RCL, with a degree of resolution that definitively precludes an insertion of even four fewer residues. In addition, our data indicate that all secondary structural elements are intact. Two further important conclusions flow from these observations: Based on RCL length, this must be incorporated in trans into a different subunit to that of the C-terminus, and any putative “second” intermolecular linkage cannot involve loss of a structural element. In combination, for both the liver-derived and heat-induced polymers, these data are inconsistent with the loop-sheet mechanism (*2*), the β-hairpin polymerization mechanism that requires unwinding of helix I (*26*), and models that have been proposed based on edge-strand interactions without RCL incorporation (*27*, *28*, *62*). Other permutations of a “double-linkage” model that could feasibly generate linear chains of polymers with repeating 60- to 70-Å subunits (*2*, *4*) are not possible without unfolding of core structured regions. Thus, only a single linkage via intermolecular exchange of the C-terminus with an adjacent molecule appears compatible with our data.

We have shown that Z AAT polymerization arising from perturbation of the native conformation in vitro, or during folding either in cells or in a mouse model of AATD, can be prevented by the interaction of a small molecule with a near-native, non–RCL-incorporated conformation of the protein (*42*). Thus, the polymerization pathway proceeds from a well-structured conformation that is attainable both by slight perturbation of the native state and during folding of the molecule. Furthermore, while during folding on the ribosome the Z AAT variant shows prolonged exposure of its C-terminus (*63*), in vitro experiments using peptide fragments to elucidate order of serpin folding showed that for self-incorporation of the RCL to occur the C-terminus must be present in its cognate position (*64*). It has additionally been established that once Z AAT has adopted a monomeric, native state, it is stable under physiological conditions and at high concentrations (*5*, *25*, *65*), and polymers show extreme stability. When considered in combination with conclusions presented here that both heat-induced and liver-derived polymers have an identical structure mediated by a single C-terminal linkage, it follows that the decision point dictating a monomeric or polymeric molecular fate is most likely the incorporation of the C-terminus in cis or in trans, which precedes eventual self-incorporation of the RCL within the recipient molecule.

In summary, the integrative structural approach used here has allowed the first characterization of the internal structure of a serpin polymer formed in human tissue in association with a serpinopathy. The hierarchical approach of assignment transfer from high-resolution recombinant reference samples to ^1^H methyl spectra demonstrates the feasibility of obtaining structural information from non-ideal samples with natural abundance, high molecular weight, and low availability. This approach may have broader applications to various systems where isotopic labeling is not feasible. Advancements in spectrometer field strengths and pulse sequence designs makes the analysis of unlabeled biomolecules more accessible. For example, this has been applied to patient-derived samples like plasma AAT (*25*), and protein therapeutics, such as mapping of monoclonal antibodies (*66*).

This work conclusively settles the conformation of heat-induced AAT polymers to be mediated by an asymmetric C-terminal intermolecular linkage capable of self-propagation. These findings were facilitated by integrating crystal structures and dynamics, as reported by NMR spectroscopy, to discriminate differences in structures that were ostensibly identical. At a residue-specific level, the data show that that this linkage is central to the polymers that deposit in liver tissue, providing a consistent basis for interpreting the molecular shape of the repeating dimeric subunit of liver-derived material (*4*). These observations are additionally consistent with a pathogenic aggregation mechanism that recapitulates a folded state encountered during normal function of the protein—that of the fully RCL-incorporated cleaved conformation. Thus, the energetic barrier that the Z variant overcomes during production in hepatocytes is not one between native and non-native but between functional start and end states, and explains the inability of the cellular machinery to recognize the resulting polymers as misfolded.

The structural characterization of AAT polymers presents the opportunity to improve diagnosis of AATD by guiding polymer-specific diagnostic development. Our contribution to understanding the pathological mechanism of AAT aggregation in liver tissue opens prospects for therapeutic strategies to target the polymerization pathway that underlies not only AATD but also potentially other aggregation-induced serpinopathies.

## MATERIALS AND METHODS

### Preparation of plasma M and Z AAT

AAT was collected from the blood of consenting donors in the presence of 10% (w/v) sodium citrate anticoagulant. Plasma was isolated by centrifugation at 3200*g* for 30 min at 4°C. The supernatant containing blood plasma was removed and centrifuged again. Plasma was frozen at −80°C until required. Defrosted samples were centrifuged at 3200*g* for 30 min at 4°C to remove aggregates, filtered through a glass wool fiber pre-filter and 0.45-μm filter, and loaded onto a column containing Alpha-1-Antitrypsin-Select resin (Cytiva) equilibrated in 20 mM tris (pH 7.4), 150 mM NaCl. After loading, the column was washed with 10 column volumes of equilibration buffer and eluted with the addition of 2 M MgCl_2_. Fractions containing AAT were dialyzed at 4°C for 16 hours in 20 mM tris (pH 8.0), 50 mM NaCl, 0.02% (w/v) NaN_3_, reduced for 10 min in 100 mM 2-mercaptoethanol, loaded onto a HiTrap Q HP ion exchange column (Cytiva), and eluted over a gradient of 0 to 1 M NaCl. Fractions containing AAT were further purified by size exclusion chromatography into 25 mM sodium phosphate (pH 8.0), 50 mM NaCl, 0.02% (w/v) NaN_3_.

Samples were from patients attending a specialized AATD clinic at the Royal Free Hospital, London. Ethics oversight: REC reference 13/LO/1085—NHS Health Research Authority NRES Committee London-Hampstead.

### Preparation of recombinant AAT

BL21 cells (New England BioLabs, Ipswich MA, USA) were cotransformed with a pQE30 vector containing the wild-type AAT open reading frame (ORF) and a pREP4 plasmid containing the *lac* repressor. Nonisotopically labeled AAT were grown in Terrific broth medium (Sigma-Aldrich) until they reached an OD_600_ (optical density at 600 nm) ~ 0.8 to 1, after which isopropyl-β-d-thiogalactopyranoside (IPTG) was added to a final concentration of 1 mM to induce protein expression. The cultures were grown a further 24 to 36 hours at 24°C with shaking at 200 rpm. The cells were harvested by centrifugation at 3200*g* at 4°C for 30 min, whereupon the pellets were resuspended in minimal volume of phosphate-buffered saline (PBS). The lysates underwent multiple freeze-thaw cycles at −80°C with lysozyme (3 mg/ml) from chicken egg white (Merck) and 0.1 mM 4-(2-aminoethyl) benzene sulfonyl fluoride hydrochloride (AEBSF) (Merck), and the appropriate concentration of Pierce protease inhibitor tablets (Thermo Fisher Scientific) was added to each lysate before sonication with a Soniprep 150 sonicator with amplitude 10 μm for 30 cycles of 15 s on and 20 s off.

Soluble protein was loaded onto a Ni-NTA charged HiTrap chelating column (Cytiva) equilibrated in 20 mM tris (pH 8.0), 150 mM NaCl, 15 mM imidazole, 0.02% (w/v) NaN_3_. After washing with five column volumes of equilibration buffer, the sample was eluted with a gradient of 15 to 300 mM imidazole. Subsequent IEC and size exclusion chromatography were conducted as described above.

Uniformly ^13^C,^15^N-labeled AAT were initially grown in BL21 cells to stationary phase in LB medium (Sigma-Aldrich). Cells were collected by centrifugation at 3200*g* for 20 min at 4°C and resuspended in EM9 medium (*67*) at OD_600_ 0.01, containing ^13^C-glucose [0.2% (w/v)] and ^15^NH_4_Cl [0.1% (w/v)]. Cells were grown to OD_600_ ~ 0.6, induced with 1 mM IPTG, and expressed for 16 hours at 24°C, 220 rpm. Cells were harvested and purified as described above.

Uniform ^2^H-Ileδ1, Leuδ1/2, Valγ1/2-[^13^CH_3_] (ILV)–labeled samples were prepared by slowly adapting cells to deuterated medium and labeled isotopes by stepwise inoculation. A single transformed colony was introduced to a 5-ml LB starter culture and grown at 37°C, shaking at 220 rpm for 3 to 5 hours. This culture was used to inoculate 5 ml of EM9 medium prepared in 80% D_2_O at OD_600_ ~ 0.1 to 0.2 and incubated overnight at 37°C, shaking at 220 rpm. Another 5 ml of EM9 medium (*67*) prepared in 100% D_2_O was inoculated and grown for 5 to 7 hours at 37°C with shaking. The next inoculated 5 ml of EM9 medium was prepared in 100% D_2_O containing 50% ^12^C-d_7_-glucose [0.1% (w/v)] and 50% unlabeled glucose [0.1% (w/v)] and grown overnight, followed by the final inoculation of EM9 medium prepared in 100% D_2_O containing ^12^C-d_7_-glucose [0.2% (w/v)] and grown at 37°C with shaking throughout the day. This culture was then used to inoculate 50 ml of EM9 medium prepared in 100% D_2_O containing ^12^C-d_7_-glucose [0.2% (w/v)] and grown at 37°C with shaking overnight. The cells were centrifuged at 3200*g* for 20 min at room temperature (JA 8.1 rotor, Beckman Coulter) and resuspended into 1 liter of fresh EM9 medium prepared with 100% D_2_O containing ^12^C-d_7_-glucose [0.2% (w/v)]. The culture was grown at 37°C with shaking until an OD_600_ ~ 0.6 was reached, at which point the temperature was reduced to 30°C and 80 mg/liter of the isoleucine precursor 2-ketobutyric acid sodium salt (methyl-^13^C, 99%; 3,3-d2) and 160 mg/liter of the leucine/valine precursor 2-ketoisovaleric acid sodium salt (3-methyl-^13^C, 99%; 3,4,4,4-d4) were added to produce methyl-labeled samples. The culture was grown for another hour and then induced with 1 mM IPTG (prepared in D_2_O) and incubated for 16 hours at 24°C. Cells were harvested by centrifugation and purified as described above. Samples were buffer-exchanged into deuterated buffer for NMR collection.

### Purification of Z AAT from explanted liver

Sections (~150 g) of explanted liver tissue Z AAT from homozygotes who had undergone transplantation for Z AATD–associated cirrhosis were thinly sliced and ground on ice using a 50-ml CS1 Tissue Grinder (DWK Life Sciences), and incubated at 37°C at 200 rpm for 1 hour in 300 ml of Hanks’ balanced salt solution (140 mM NaCl, 5 mM KCl, 1 mM CaCl_2_, 0.4 mM MgSO_4_, 0.3 mM Na_2_HPO_4_, 6 mM glucose, 4 mM NaHCO_3_) with 15 mg of collagenase (type 1A, Merck). The digested sample was filtered through nylon mesh, and the filtrate was centrifuged at 4°C for 30 min at 3000*g*. The pellet was resuspended in 5 ml of filtered PBS, sonicated (Q500 Sonicator, QSonica) in a chilled water bath at 50% amplitude for 60 s, applied on top of 500 ml of 1.3 M sucrose at 4°C, and centrifuged at 16,000*g* at 4°C for 1 hour. The supernatant was discarded, and the pellet was repeatedly washed in PBS by resuspension and centrifugation for 15 min at 16,000*g* and 4°C until the supernatant appeared clear.

Soluble polymers were released from the resulting inclusion bodies by sonication in a chilled water bath at 50% amplitude for 15 s on/30 s off for 10 min, with non-extractable components removed by repeated resuspension and centrifugation at 16,000*g* at 4°C for 15 min. The components were filtered (0.45 μm) and loaded onto a 5-ml HiTrap Chelating HP column (Cytiva Biosciences) charged with CuSO_4_ and pre-equilibrated in buffer A [20 mM Na_2_HPO_4_ (pH 7.4), 500 mM NaCl, 0.02% (w/v) NaN_3_]. The column was washed with 10 column volumes of buffer A before elution with a 0 to 100 mM imidazole gradient. Following SDS-PAGE gel evaluation, fractions seemingly containing AAT were dialyzed into buffer B [20 mM tris-HCl (pH 8.0), 0.02% (w/v) NaN_3_] and loaded onto a 1-ml HiTrap Q HP column (Cytiva Biosciences) pre-equilibrated in the same buffer. Following elution with a 0 to 1 M NaCl gradient in buffer B, fractions containing AAT were purified by size exclusion chromatography on a Superdex 200 Increase 10/300 GL column (Cytiva Biosciences), dialyzed into 25 mM Na_2_HPO_4_, pH 8.0, 50 mM NaCl, 1 mM EDTA, 1 mM dithiothreitol (DTT), 0.02% (w/v) NaN_3_, 10% (v/v) D_2_O, and then stored at −80°C until required.

### Preparation of AAT polymers by heat perturbation

AAT polymers were induced in vitro by heating AAT (0.2 mg/ml) at 55°C for 48 hours in a water bath. Following confirmation of polymerization by nondenaturing PAGE, the sample was purified using a HiTrap Q Sepharose column (Cytiva) with a 0 to 1 M NaCl gradient in 20 mM tris (pH 8.0), 0.02% (w/v) NaN_3_ to separate the polymeric AAT from residual monomer (*4*).

### Preparation of cleaved AAT

Cleaved AAT was generated by incubating the protein in a stoichiometric excess ratio of 1:100 with endoproteinase Glu-C (Merck) or 1:1.5 with bovine chymotrypsin (Sigma-Aldrich) in a water bath overnight at 37°C. After incubation, protease inhibitor tablets (Pierce) were added to quench the reactions. The samples were loaded onto a 1-ml HiTrap Q HP anion exchange chromatography column (Cytiva Biosciences) pre-equilibrated in 20 mM tris-HCl (pH 8.0), 0.02% (w/v) NaN_3_ and eluted with a 0 to 1 M NaCl gradient to separate the uncleaved material from the cleaved AAT.

### Sample analysis by electrophoresis

Protein was characterized under denaturing conditions by the NuPAGE bis-tris gel system (Life Technologies). Typically, 4 μg of protein was mixed with NuPAGE lithium dodecyl sulfate (LDS) sample buffer (1× final concentration) containing DTT (150 mM final concentration) and heated to 90°C for 4 min. Samples were resolved using 4 to 12% (w/v) acrylamide bis-tris SDS-PAGE gels at 180 V for 52 min in NuPAGE Mops-SDS running buffer (Life Technologies). The molecular weights of sample components were estimated by comparison to the Spectra Multicolour Broad Range protein ladder (Thermo Fisher Scientific).

Protein characterized under nondenaturing conditions used the Novex bis-tris 3 to 12% (w/v) acrylamide gel system (Life Technologies). Samples were mixed 1:1 with sample buffer [50% (v/v) glycerol containing bromophenol blue] and loaded 4 μg of protein per lane. Running conditions were constant at 50 mA per gel for 22 min in 1× Native PAGE running buffer (Life Technologies). Gels were visualized by staining with Instant blue Coomassie stain (Expedeon).

### NMR spectroscopy

NMR experiments were performed on 700 MHz Bruker Avance III HD, 800 MHz Bruker Avance III HD, 900 MHz Bruker Avance III, 950 MHz Bruker Avance III, and 1000 MHz Bruker Avance III HD spectrometers, all equipped with TXI or TCI cryogenic probes and uniaxial gradient coils generating a maximum gradient strength of approximately 0.55 T m^−1^.

Isotopically labeled, recombinant AAT samples were prepared in 25 mM Na_2_HPO_4_, pD 7.6, 50 mM NaCl, 1 mM EDTA, 1 mM DTT, 0.02% (w/v) NaN_3_, in 100% (v/v) D_2_O. Ex vivo AAT samples were in 25 mM Na_2_HPO_4_ (pH 8.0), 50 mM NaCl, 1 mM EDTA, 1 mM DTT, 0.02% (w/v) NaN_3_, in 10% (v/v) D_2_O. All NMR experiments were recorded using 5-mm-diameter Shigemi tubes or 5-mm Bruker tubes, and in a range of 298 to 328 K, depending on the nature of the sample and experiment. Temperature calibration was performed using the chemical shift difference of perdeuterated methanol as a function of temperature as described by Karschin *et al.* (*68*). All NMR spectra were processed with nmrPipe and imported into CCPN Analysis or MATLAB (R2020b, The MathWorks Inc.) for analysis.

For recombinant samples, typical 1D ^1^H spectra were acquired using excitation sculpting for water suppression, with spectral widths of 20 ppm and 32,768 points for an acquisition time of ~1.14 s, and a recycle delay of 1 s. For ex vivo samples, typical 1D ^1^H spectra were acquired using excitation sculpting for water suppression, with spectral widths of 16 ppm and 32,768 complex points for an acquisition time of ~1.14 s, and a recycle delay of 1 s. Spectra were processed using cosine window functions.

2D ^1^H,^13^C methyl-TROSY-HMQC spectra were acquired using 256 points with a sweep width of 17.5 ppm in the indirect ^13^C dimension, and 4096 points and a sweep width of 20 ppm in the direct ^1^H dimension, corresponding to acquisition times of ~32 ms and ~114 ms, respectively. Spectra were recorded with a 1-s inter-scan delay. Spectra were processed using cosine squared window functions.

### Diffusion measurements

^1^H stimulated echo (STE)-HMQC experiments were recorded with bipolar SMSQ10.100 gradients with a total encoding/decoding gradient length, δ, of 4 ms, a diffusion delay, Δ, of 75 ms, and 16 points at gradient strengths between 5% and 95% of the maximum, approximately 55 G cm^−1^. The signal was integrated at each gradient strength, and the diffusion coefficient was calculated using Stejskal-Tanner equation by nonlinear fitting. Error bars report the standard error of the diffusion coefficient determined from the nonlinear fit. The hydrodynamic radius was estimated from the measured diffusion coefficient and known viscosity of D_2_O (*69*) at 328 K using the Stokes-Einstein equation.

### Assignment of cleaved AAT spectra

Because of the thermostability of the cleaved state, assignment experiments could be acquired at high temperature (321 K) without sample degradation. At this temperature, solution viscosity is approximately half that at room temperature, which enabled assignment of the relatively high molecular weight molecule without requirement for deuteration. Backbone resonance assignment was performed using BEST-TROSY, HNCO, HNCA, HNCACO, HNCOCA, HNCOCACB, and HNCACB experiments (*70*, *71*) were acquired with a recycle delay of 300 ms and typically 100-ms acquisition times in the ^1^H dimension, 48 complex points and a 32-ppm spectral width in the ^15^N dimension, 48 complex points and a 12-ppm spectral width in ^13^CO dimensions, 48 complex points and a 30-ppm spectral width in ^13^CA dimensions, and 128 complex points and a 75-ppm spectral width for ^13^CA/B dimensions. The I-PINE web server (*72*) was used to generate an initial assignment that was manually validated. The completed assignment covered 91% of ILV backbone residues for the N-terminal fragments and 69% for the C-terminal fragment. Unassigned backbone resonances were mainly located in loops, which we attribute to rapid amide exchange broadening at high temperatures. Side-chain assignment was then performed using a TROSY HBHACONH experiment, with 36 complex points and a 5-ppm spectral width in the ^1^HA/B dimension; a CC(CO)NH TOCSY experiment (*73*), with 128 complex points and a 75-ppm spectral width in the ^13^C dimension; and HCH and CCH COSY and TOCSY experiments (*74*), with 128 complex points and a 70-ppm spectral width in ^13^C dimensions, and 128 complex points and a 7.2-ppm spectral width in the indirect ^1^H dimension. Stereospecific assignments of leucine and valine methyl resonances were determined by comparison with a stereospecific [^2^H, A^β^I^δ1^L^δ2^V^γ2^M^ε^-^13^CH_3_]–labeled sample of cleaved AAT. Ninety-four percent of δ1 and γ2 Ile resonances, 85% of γ1/γ2 Val resonances, and 74% of the δ1/δ2 Leu resonances were assigned for the N-terminal fragment, and 33% of Ile δ1 and γ2, 25% of Val γ1, and 33% of Leu δ1 resonances were assigned for the C-terminal fragment (fig. S4).

### Attribution of polymer spectra

AAT monomer resonances have previously been attributed by transfer of assignments from isotopically labeled recombinant wild type following a conservative, iterative strategy (*25*). The same approach was used here to transfer the assignments of cleaved AAT to heat-induced AAT polymer. The cleaved and polymer spectra were recorded in the same experimental conditions to ensure that no resonance perturbations were the result of nonstructural effects, such as pH or temperature. This allowed assignments to be transferred to polymer resonances, initially within a threshold of 0.05 ppm (^1^H) CSPs. Any further resonance shifts were transferred if they could be justified by known structural differences between the cleaved AAT [Protein Data Bank (PDB): 1EZX (*51*)] and C-terminal circular trimer crystal structures [PDB: 3T1P (*37*)] that best represent the circular polymer structures seen from EM.

The assignments of methyl resonances for ^1^H 1D spectra were attributed using a similar approach as stated above. The NMR data for recombinant and plasma M and Z AAT (cleaved at the RCL with chymotrypsin and Glu-C) and liver-derived LLO polymers were collected under identical conditions [328 K, 25 mM Na_2_HPO_4_ (pH 8.0), 50 mM NaCl, 1 mM EDTA, 1 mM DTT, 0.02% (w/v) NaN_3_, 10% (v/v) D_2_O]. The ^1^H methyl assignments from ^1^H,^13^C HMQC spectra of recombinant M AAT cleaved with Glu-C and chymotrypsin were transferred to the corresponding ^1^H plasma AAT resonances with the same conservative, iterative approach as previously used to account for known slight shifts associated with glycosylation and the E342K mutation (*25*). The ^1^H spectra of Glu-C and chymotrypsin-cleaved plasma protein differed in the chemical shifts of a number of residues (V321, V311, V173, and V145), which were also shifted in their 2D recombinant counterparts. The plasma chymotrypsin-cleaved AAT spectrum overlaid near-identically with the LLO liver polymer spectrum. The peaks in the chymotrypsin-cleaved plasma spectrum that did not correspond with plasma cleaved with Glu-C were also absent for LLO liver polymer, suggesting full RCL insertion. The LLO liver polymer peaks were assigned by transferring assignments from plasma Z AAT cleaved with chymotrypsin at identical chemical shift (Table S2).

### NS-EM of AAT polymers

Copper grids (400 mesh) with a continuous carbon support (Electron Microscopy Sciences) were glow-discharged for 1 min at 30 mA (Ted Pella). Three microliters of AAT polymers at 0.05 mg/ml were deposited onto the grids and negatively stained using 2% uranyl acetate (w/v) as previously described (*4*). The grids were visualized using an FEI Tecnai T12 microscope with a LaB_6_ source operating at 120 keV, and images were recorded on a Gatan CCD MultiScan camera at ×67,000 nominal magnification and 2-μm defocus.

### X-ray crystallography

2C1 Fab fragments were generated using the Pierce Fab Preparation Kit (Thermo Fisher Scientific) and incubated with Glu-C–cleaved AAT for 1 hour at room temperature. Crystals were obtained from sitting drops containing 0.2 M ammonium citrate dibasic, 20% (w/v) polyethylene glycol 3,350 (pH 5.1) at 293 K. Crystals mounted on nylon loops were briefly soaked in the crystallization buffer and cryoprotected in 10% (v/v) ethylene glycol before plunge-freezing into liquid nitrogen. Data collection was undertaken at the European Synchrotron Radiation Facility (ESRF) ID23-2 beamline. Data merging and scaling was performed by Aimless (*75*) and reduction by XDS (*76*). The structure was solved by molecular replacement using Phaser (*77*) and model refinement using PHENIX (*78*), and model visualization and building were performed in Coot (*79*).
