# Supplementary material for: High-resolution characterization of ex vivo AAT polymers by solution-state NMR spectroscopy
Source: Sci Adv. 2025 May 7;11(19):eadu7064. doi: 10.1126/sciadv.adu7064 (PMC12057664; doi:10.1126/sciadv.adu7064)
Supplement: Supplementary file 1 — Figs. S1 to S12 Tables S1 and S2 [file sciadv.adu7064_sm.pdf]

Science Advances
AAAS

Supplementary Materials for

High-resolution characterization of ex vivo AAT polymers by solution-state NMR spectroscopy

Sarah M. Lowen *et al.*

Corresponding author: Sarah M. Lowen, sarah.lowen.20@ucl.ac.uk; Christopher A. Waudby, c.waudby@ucl.ac.uk; James A. Irving, j.irving@ucl.ac.uk

Sci. Adv. **11**, eadu7064 (2025)
DOI: 10.1126/sciadv.adu7064

This PDF file includes:

Figs. S1 to S12
Tables S1 and S2

Fig. S1.

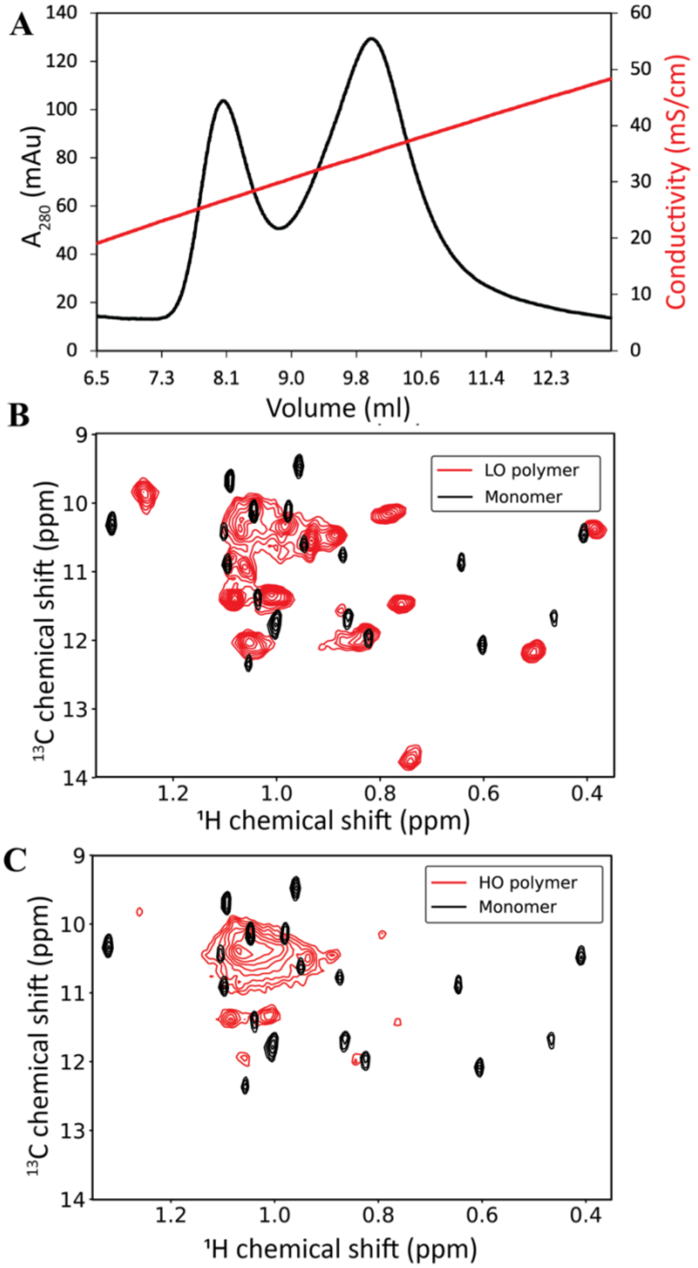

Comparison of lower-order and higher-order heat-induced polymers. A) Ion-exchange chromatography purification of heat-polymerised AAT to separate two polymer species. Ile region of ^{1}H -^{13}C HMQC spectra overlay of monomer AAT, B) LO polymer and C) HO polymer with ^{13}C-methyl-ILV labelling (328K, 950MHz). Contour levels normalised for concentration.

Fig. S2.

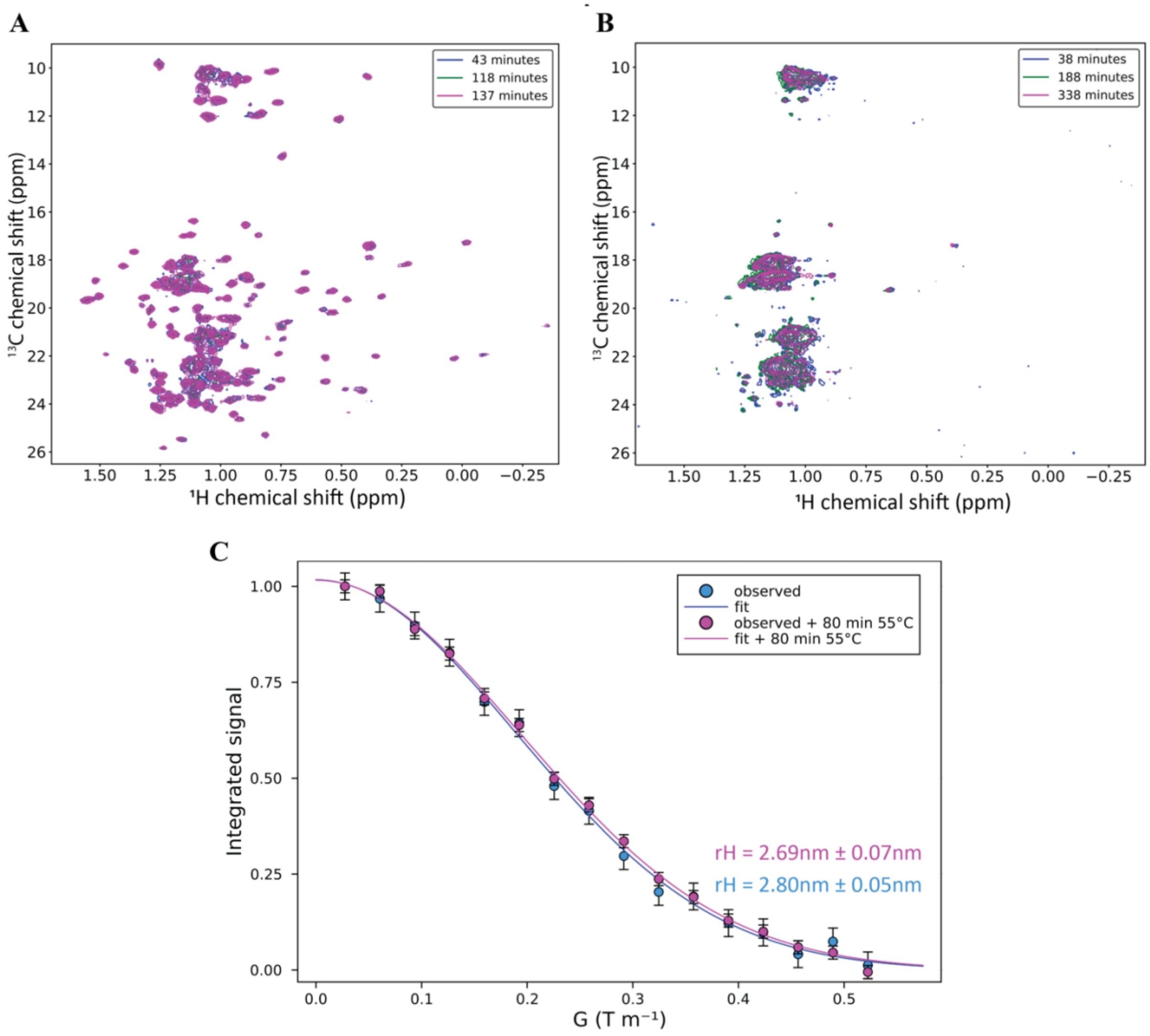

Lower-order and higher-order heat polymers did not undergo temperature-induced changes during data collection at 328K. A) Overlaid ^{1}H, ^{13}C HMQC spectra of ^{13}C-methyl-ILV labelled lower-order and B) higher-order heat polymers with cumulative time at 328K during data collection. C) ^{1}H STE-HMQC translational diffusion data of lower-order polymer at initial acquisition and after 80 minutes at 328K. Observed fit calculated using the Stejskal-Tanner equation and apparent hydrodynamic radius calculated using the Stokes-Einstein equation.

Fig. S3.

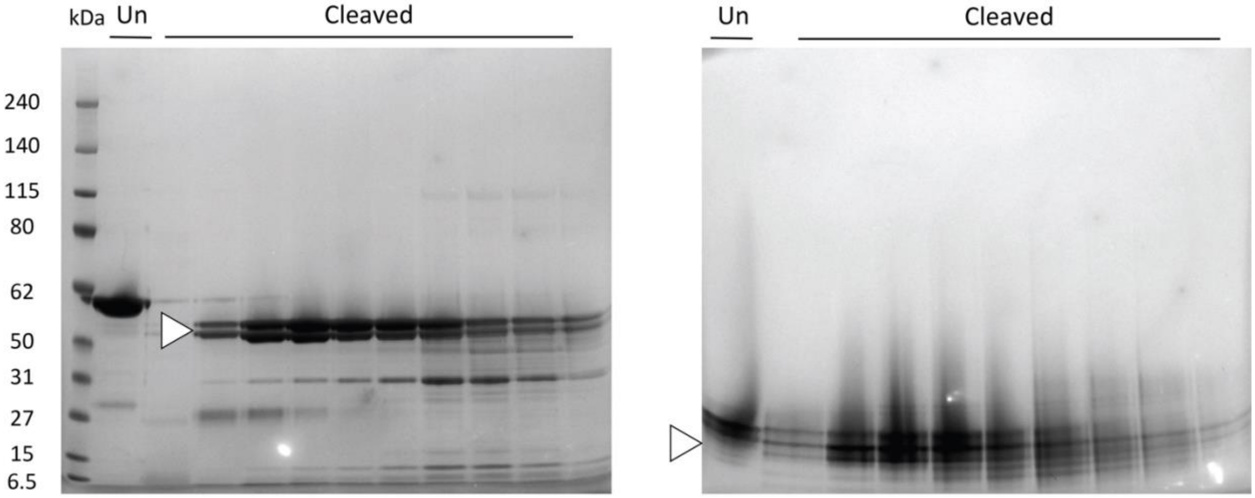

4-12% (w/v) acrylamide SDS and 3-12% (w/v) acrylamide non-denaturing PAGE of AAT cleaved by Glu-C and purified by ion exchange chromatography. The lanes represent sequential fractions from the ion exchange column. Bands at expected positions for cleaved AAT are indicated with a white arrow. Uncleaved, native AAT provided a reference in both gels (Un).

Fig. S4.

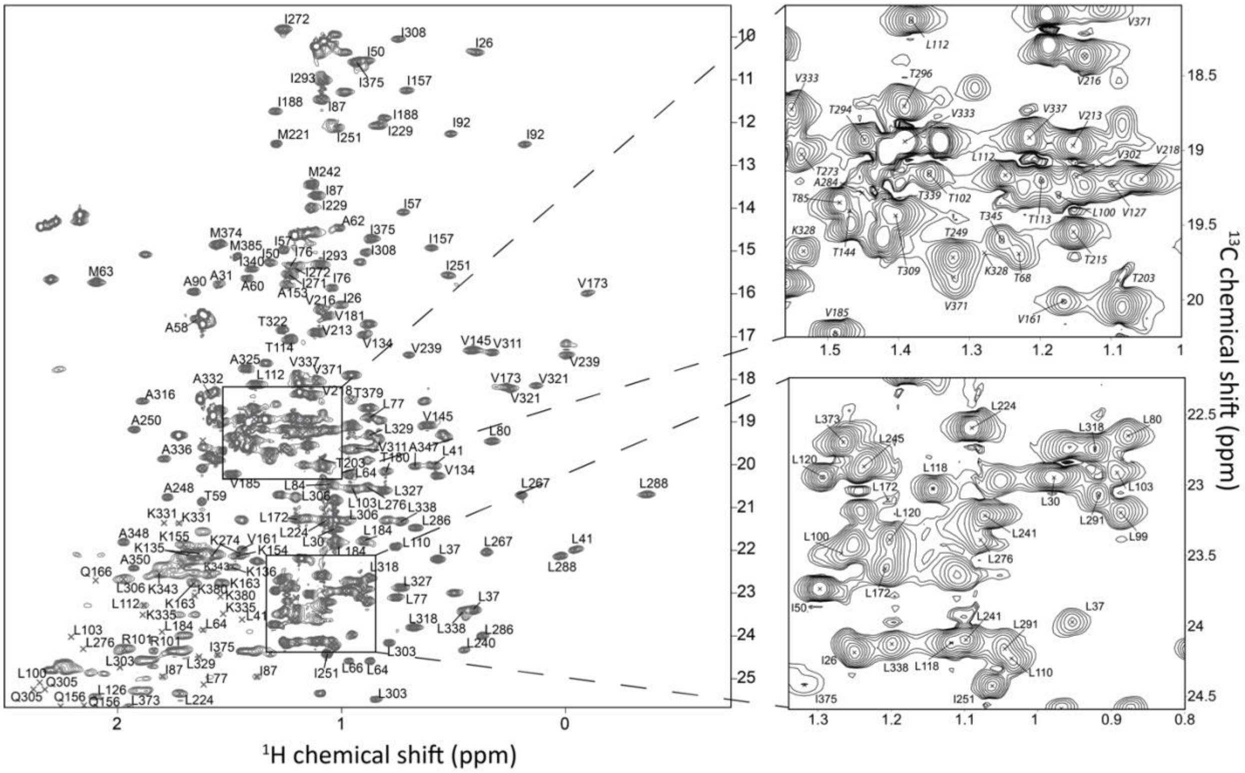

The methyl region assignment of AAT cleaved at the reactive centre loop with Glu-C. Assignment of ¹H ¹³C HSQC spectrum of 240μM ¹³C, ¹⁵N-labelled cleaved AAT in 25mM sodium phosphate, 50mM NaCl, 1mM EDTA, 10% v/v D_2O, pH 8.0, acquired at 321K on a Bruker 700MHz cryoprobe. Assignments are in the format of residue position in the sequence according to the 9GGP crystal structure, followed by the residue type letter. The full methyl region (*left*) and close-ups of the regions of resonance overlap (*right)*. BMRB ID: 52599.

Fig. S5.

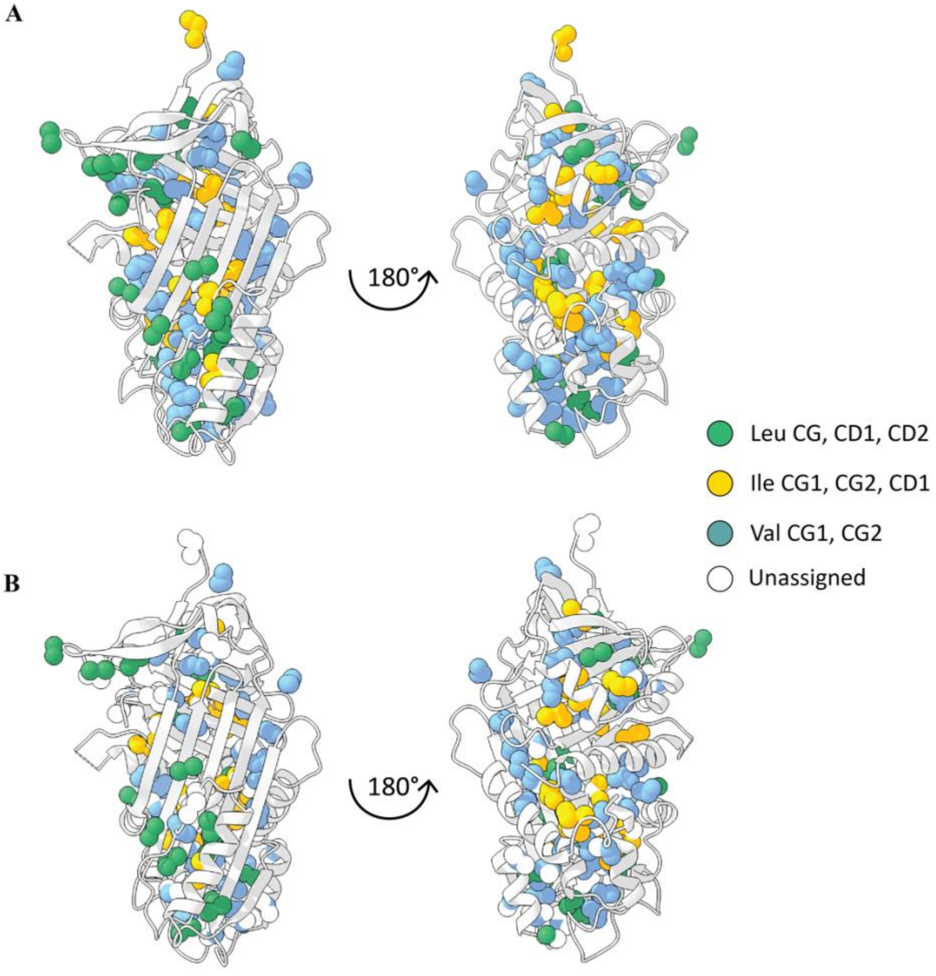

Final methyl assignment coverage of ILV AAT residues. Cartoon representation of cleaved AAT [PDB: 9GGP; reported here] with A) total and B) assigned Leu Cγ Cδ1/2 (green), Ile Cγ1/2 Cδ1 (yellow), and Val Cγ1/2 (blue) residues shown as spheres. Unassigned methyl resonances are shown as white spheres.

Fig. S6.

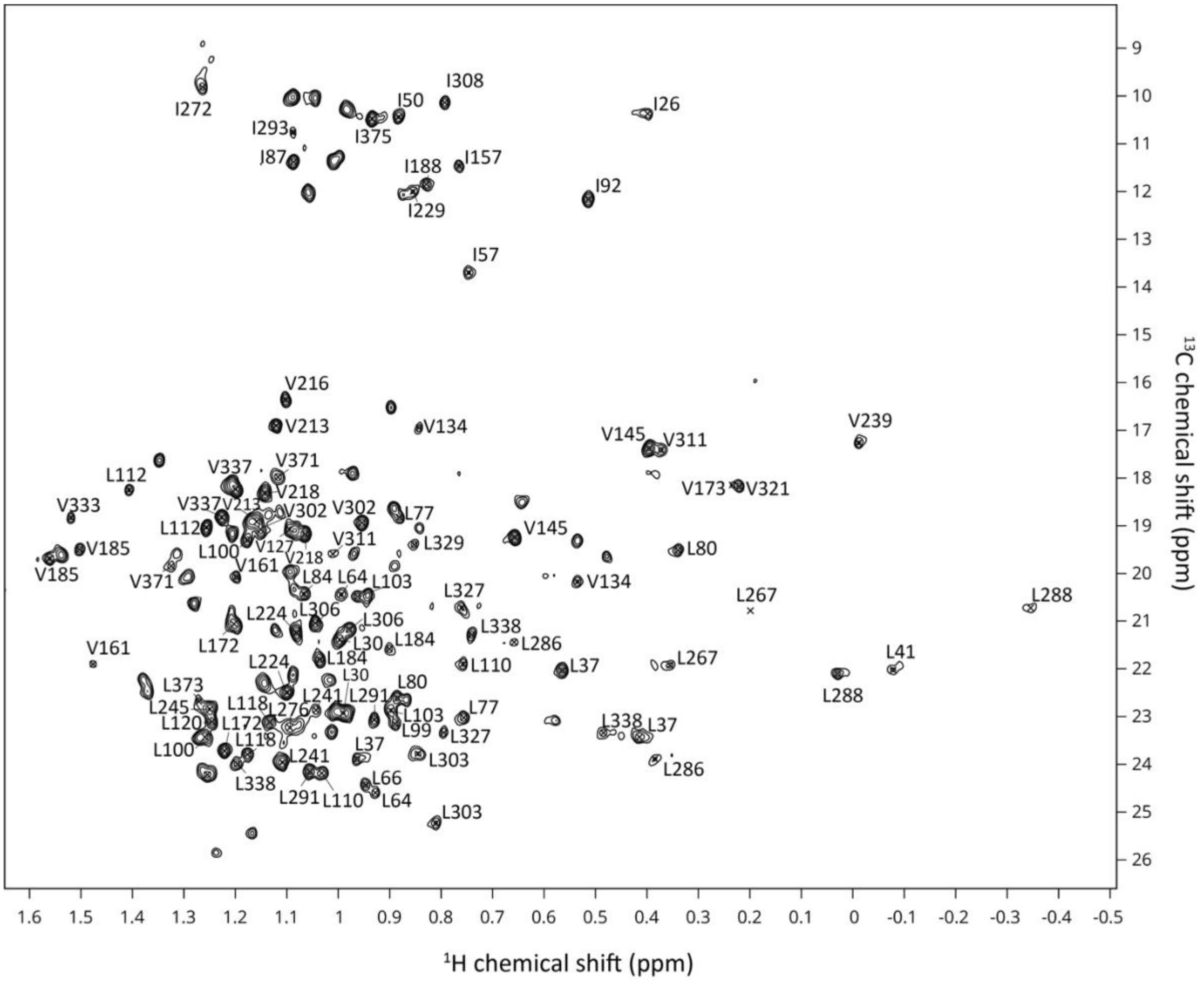

Assignment of ILV isotopically labelled AAT cleaved at the reactive centre loop with chymotrypsin. Assignment of ^{1}H ^{13}C HMQC spectrum of 1.7µM ^{13}C-methyl-ILV labelled cleaved AAT in 25mM sodium phosphate, 50mM NaCl, 1mM EDTA, 100% v/v D_2O, pH* 7.6, acquired at 328K on a Bruker 950MHz cryoprobe. Assignments are denoted according to residue numbering in the sequence according to the 9GGP crystal structure.

Fig S7.

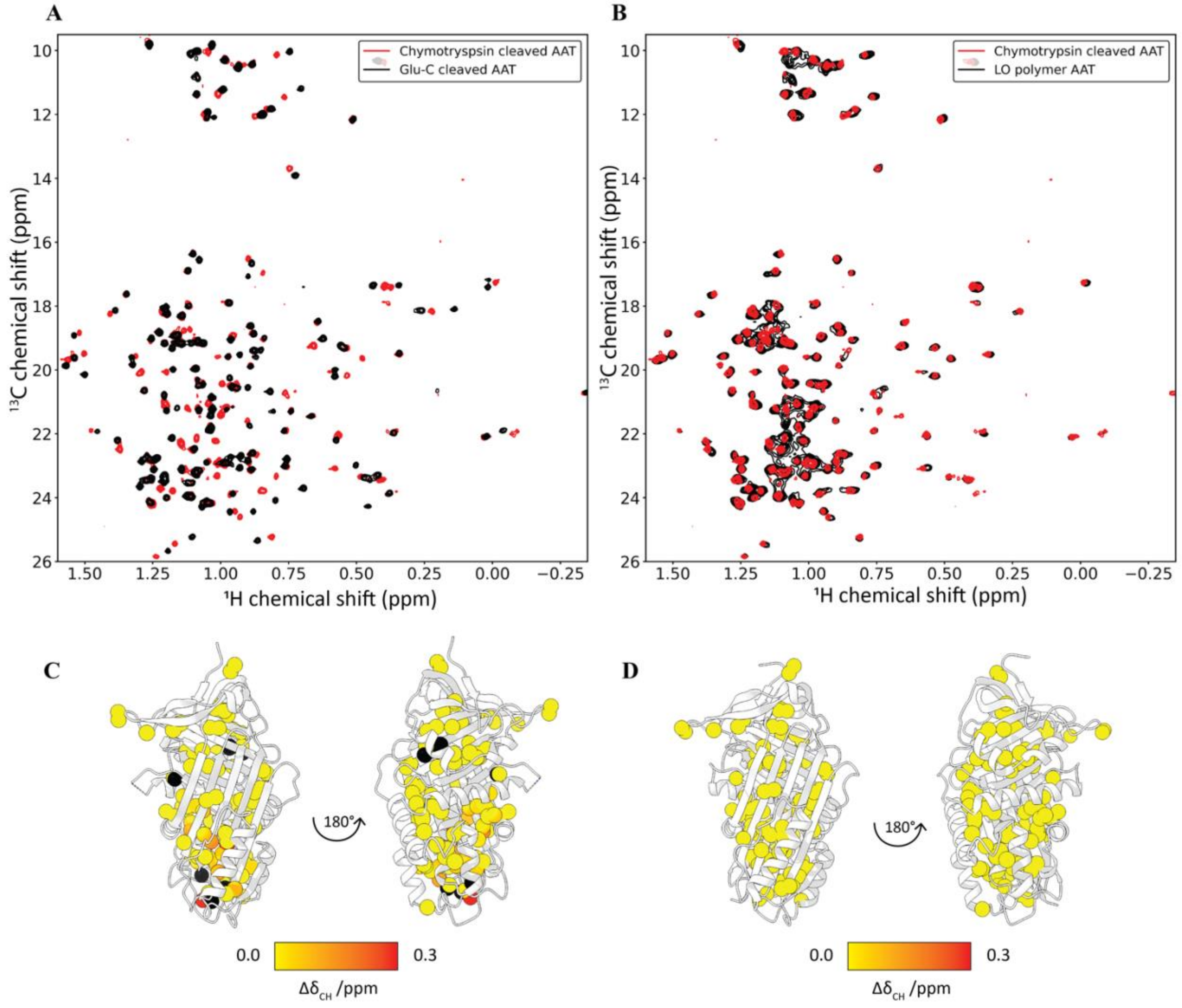

Comparison of lower-order heat-induced polymer and chymotrypsin-cleaved AAT. ^{1}H -^{13}C HMQC spectra overlay of chymotrypsin cleaved AAT (red) with (A) Glu-C cleaved AAT (black) and (B) LO heat polymer (black). Collected at 328K, 950MHz. Contour levels normalised for concentration. (C, D) Combined methyl shift changes, $\Delta\delta_{CH} = \sqrt{\Delta\delta^{2}_{H} + \left(\frac{\Delta\delta_{C}}{4}\right)^{2}}$, of A and B, respectively, projected onto the fully inserted RCL cleaved AAT structure [PDB: 1EZX (*51*)] with observed methyl groups shown as coloured spheres representing the chemical shift perturbations with a gradient from Δδ 0.0ppm (yellow) to Δδ 0.3ppm (red). Black spheres are resonances that could not be reliably transferred due to spectral overlap or drastic chemical shifts.

Fig. S8.

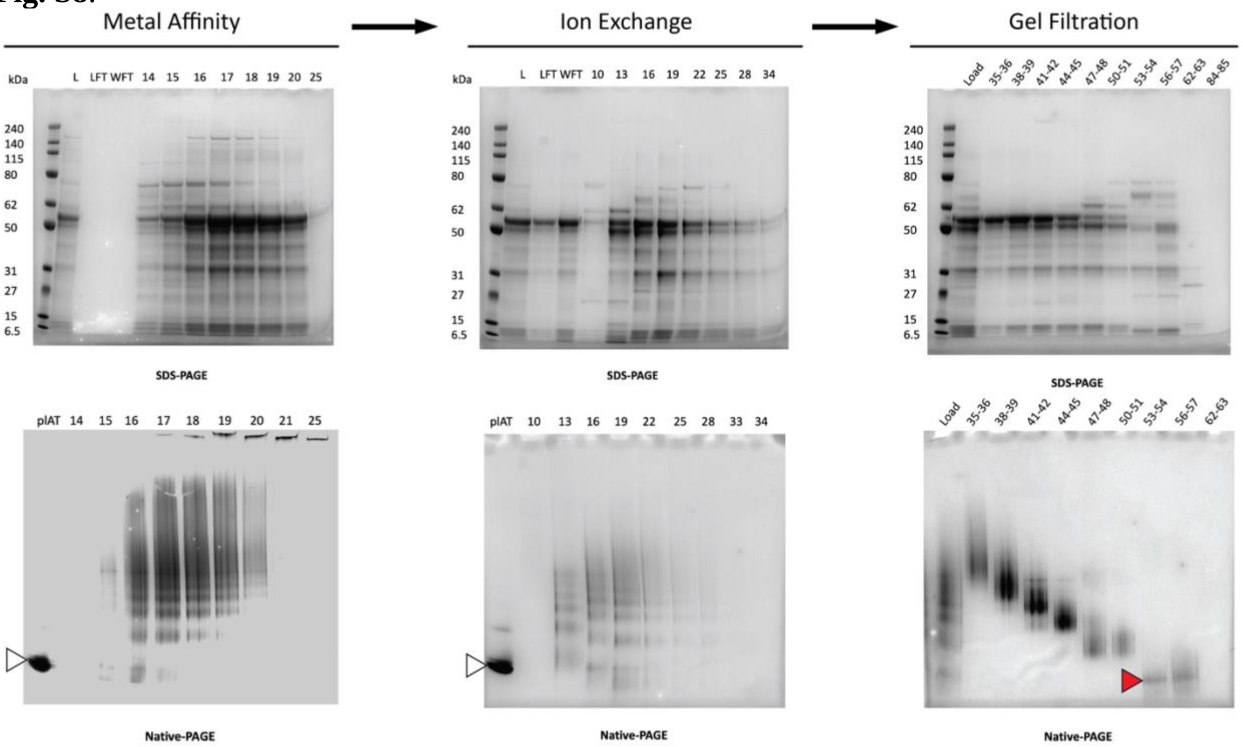

Optimised method for purifying Z AAT polymers from human liver inclusions. 4-12% (w/v) acrylamide SDS- and 3-12% (w/v) acrylamide non-denaturing-PAGE of AAT purified by sucrose centrifugation, inclusion body washes, sonication and then metal affinity chromatography. The sample was then subjected to ion exchange and gel filtration chromatography. The non-denaturing-PAGE of the gel filtered sample shows the highest level of purity of liver derived Z AAT polymers separated into different polymer sizes. White arrows indicate the migration of plasma Z AAT monomer on non-denaturing gels with the red arrow representing the liver-derived monomer.

Fig. S9.

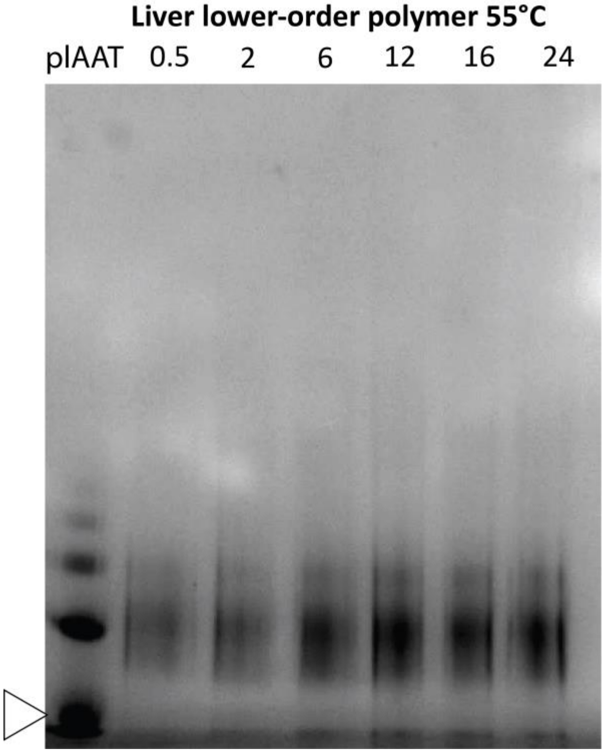

Heat stability of liver conformers. Liver-derived low-order oligomers of AAT (4µg/lane) incubated at 55ºC for the indicated time up to 24 hours, separated by 3-12% (w/v) acrylamide non-denaturing PAGE. Monomeric plasma M AAT is highlighted with a white arrow.

Fig. S10.

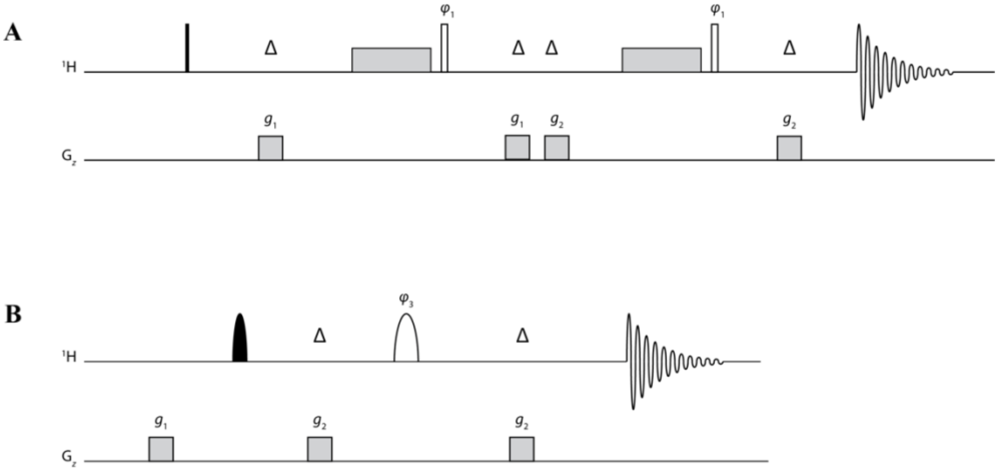

1D ^1H pulse programmes used to record the NMR spectrum of Z AAT polymers isolated from explanted liver. A) The ^1H 1D excitation sculpting (zgesgp) pulse sequence. B) The SOFAST-1D pulse sequence. The solid black shaped pulse represents a 120° PC9 pulse and the white shaped pulse is a 180° r-SNOB pulse, applied to the methyl protons at 0.5 ppm.

Fig. S11.

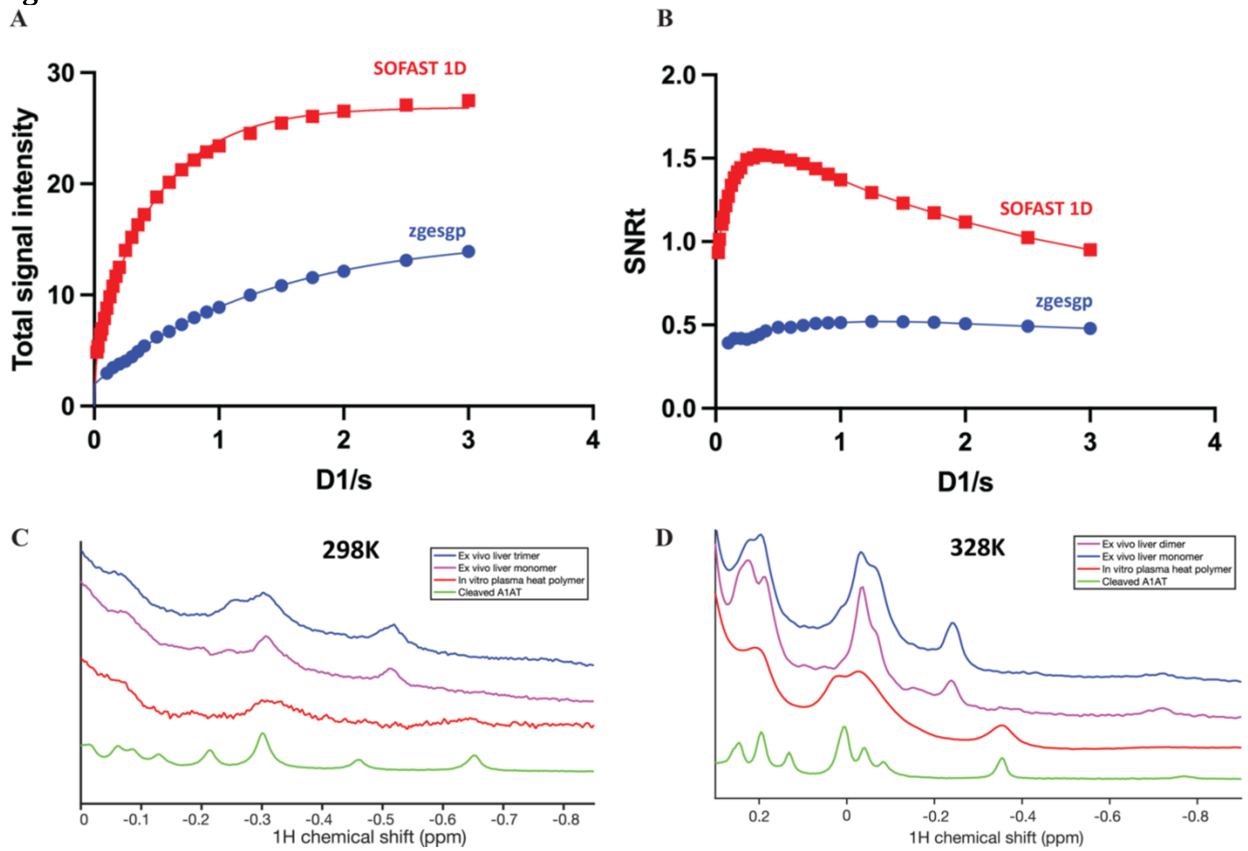

Optimisation of the SOFAST 1D pulse program and higher temperature yields a 3-fold increase in sensitivity in the methyl region. A) The total signal intensities were plotted over the D1/s, illustrating the faster recovery time for SOFAST 1D. (298K) B) The optimal D1 time, 350 ms, was calculated from observing the SNR. (298K) C) 1D NMR spectra of dimeric and monomeric Z AAT isolated from hepatic inclusions and two controls, heat induced AAT polymers and Glu-C cleaved AAT, all collected at 298K using the zgesgp pulse program. D) 1D NMR spectra of the same samples collected at 328K using the SOFAST 1D pulse program.

Fig. S12.

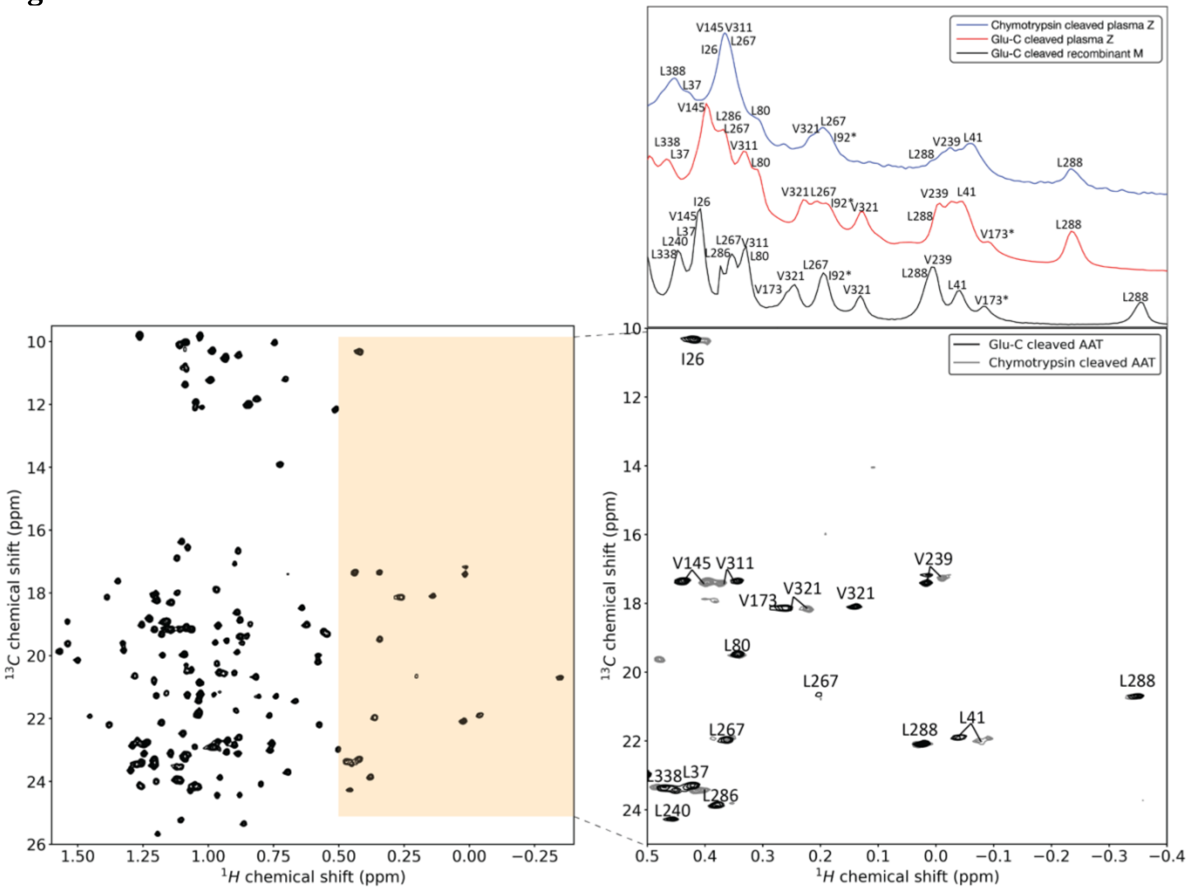

Assignment transfer from 2D spectra of recombinant samples to 1D spectra of plasma-derived samples. (Bottom left) Full ^{1}H,^{13}C HMQC spectrum of [U-^{2}H, ILVMA-$^{13}CH_3$]-labelled Glu-C cleaved AAT. The highlighted panel is enlarged on the right to show dispersion of high-field methyl resonances of Glu-C and chymotrypsin cleaved AAT. The top panel shows ^{1}H spectra of non-isotopically labelled recombinant Glu-C cleaved AAT (asterisks indicate methyl moieties at low intensity in ILV spectrum that are more visible in the ^{13}C,^{15}N HSQC spectrum (BMRB ID: 52599)). Assignment transfers from the isotopically labelled Glu-C and chymotrypsin-cleaved AAT could be made to the ^{1}H spectra of their glycosylated counterparts, with reference to marginal chemical shifts that arise from differences in glycosylated versus non-glycosylated forms, and M versus Z AAT (i.e. L288 is close to E342K mutation site) as determined previously (25). Peaks in the ^{1}H spectra of Glu-C and chymotrypsin-cleaved plasma Z AAT were largely similar, except for shifts that corresponded with those also seen in the 2D spectra of the recombinant material. A structure-guided approach was used here to cross-reference spectral differences with crystal structures (PDB: 9GGP and 1EZX), supporting chemical shift perturbations at the site of RCL-insertion into the central β-sheet (i.e. V321, V311, L41, V145, and V173).

Table S1.

**Data collection**	
Resolution range (Å)	39.90–1.84 (1.90-1.84)
Space group	P 21 21 2
Cell constants<br>a, b, c,<br>α, β, γ	 140.95Å 75.55Å 87.15Å<br>90.00° 90.00° 90.00°
Total reflections	1060725 (106307)
Unique reflections	81380 (7899)
Multiplicity	13.0 (13.5)
Completeness (%)	99.9 (100.0)
Mean I/sigma (I)	10.3 (1.1)
Wilson B-factor (Å^2)	26.0
R-merge	0.177 (2.869)
R-meas	0.184 (2.981)
R-pim	0.051 (0.805)
CC1/2	0.998 (0.564)
**Refinement**	
Reflections used in refinement	81305
Reflections used for R-free	2000 (2.46%)
R-work	0.173
R-free	0.206
Number of non-hydrogen atoms	6859
Macromolecules<br>Ligands<br>Solvent	2<br>3<br>610
Protein residues	795
RMS (bonds) (Å)	0.007
RMS (angles) (°)	0.85
Ramachandran plot (%)	
Favoured<br>Allowed<br>Outliers	98.47<br>1<br>0.13
Rotamer outliers (%)	1.59
Clashscore	1.54
B-factor (Å^2)	
Average<br>Solvent	39.0<br>49.9

Crystallographic data and refinement statistics for Glu-C cleaved AAT in complex with the Fab fragment of 2C1(PDB: 9GGP).

Table S2.

Samples		^{1}H chemical shift (ppm)										
		L288	V173	L41	V239	L267	V321	L80	L267	V311	V145	L388
Recombinant	Glu-C cleaved AAT-M	-0.347	-0.073	-0.040	0.019	0.201	0.140<br>0.261	0.339	0.360	0.344	0.439	0.467
	Chymotrypsin cleaved AAT-M	-0.347	—	-0.080	-0.015	0.201	—<br>0.222	0.339	0.360	0.373	0.396	0.467
Glycosylated	Glu-C cleaved AAT-Z	-0.234	-0.088	-0.040	-0.003	0.191<br>0.373	0.131<br>0.232	0.314	0.369	0.333	0.400	0.465
	Chymotrypsin cleaved AAT-Z	-0.234	—	-0.059	-0.021	0.191<br>0.356	—<br>0.216	0.314	0.356	0.368	0.370	0.461
	LLO polymer AAT-Z	-0.237	—	-0.061	-0.029	0.191<br>0.356	—<br>0.221	0.311	0.358	0.369	0.369	0.461

^{1}H chemical shifts (ppm) of LLO polymers, Glu-C- and chymotrypsin-cleaved recombinant and plasma AAT attributed methyl residues. Values in grey are those attributed to differences in RCL insertion (as shown in Figure 6D).
